# Methods for computing the maximum performance of computational models of fMRI responses

**DOI:** 10.1371/journal.pcbi.1006397

**Published:** 2019-03-08

**Authors:** Agustin Lage-Castellanos, Giancarlo Valente, Elia Formisano, Federico De Martino

**Affiliations:** 1 Department of Cognitive Neuroscience, Faculty of Psychology and Neuroscience, Maastricht University, Maastricht, The Netherlands; 2 Department of NeuroInformatics, Cuban Center for Neuroscience, Cuba; 3 Maastricht Centre for Systems Biology (MaCSBio), Maastricht University, Maastricht, The Netherlands; 4 Center for Magnetic Resonance Research, Department of Radiology, University of Minnesota, Minneapolis, United States of America; Western University, CANADA

## Abstract

Computational neuroimaging methods aim to predict brain responses (measured e.g. with functional magnetic resonance imaging [fMRI]) on the basis of stimulus features obtained through computational models. The accuracy of such prediction is used as an indicator of how well the model describes the computations underlying the brain function that is being considered. However, the prediction accuracy is bounded by the proportion of the variance of the brain response which is related to the measurement noise and not to the stimuli (or cognitive functions). This bound to the performance of a computational model has been referred to as the noise ceiling. In previous fMRI applications two methods have been proposed to estimate the noise ceiling based on either a split-half procedure or Monte Carlo simulations. These methods make different assumptions over the nature of the effects underlying the data, and, importantly, their relation has not been clarified yet. Here, we derive an analytical form for the noise ceiling that does not require computationally expensive simulations or a splitting procedure that reduce the amount of data. The validity of this analytical definition is proved in simulations, we show that the analytical solution results in the same estimate of the noise ceiling as the Monte Carlo method. Considering different simulated noise structure, we evaluate different estimators of the variance of the responses and their impact on the estimation of the noise ceiling. We furthermore evaluate the interplay between regularization (often used to estimate model fits to the data when the number of computational features in the model is large) and model complexity on the performance with respect to the noise ceiling. Our results indicate that when considering the variance of the responses across runs, computing the noise ceiling analytically results in similar estimates as the split half estimator and approaches the true noise ceiling under a variety of simulated noise scenarios. Finally, the methods are tested on real fMRI data acquired at 7 Tesla.

## Author summary

Encoding computational models in brain responses measured with fMRI allows testing the algorithmic representations carried out by the neural population within voxels. The accuracy of a model in predicting new responses is used as a measure of the brain validity of the computational model being tested, but the result of this analysis is determined not only by how precisely the model describes the responses but also by the quality of the data. In this article, we evaluate existing approaches to estimate the best possible accuracy that any computational model can achieve conditioned to the amount of measurement noise that is present in the experimental data (i.e. the noise ceiling). Additionally we introduce a close form estimation of the noise ceiling that does not require computationally or data expensive procedures. All the methods are compared using simulated and real fMRI data. We draw conclusions over the impact of regularization procedures and make practical recommendations on how to report the results of computational models in neuroimaging.

## Introduction

Computational modelling approaches applied to functional magnetic resonance imaging (fMRI) measurements aim to explain and predict the brain responses by expressing them as a function of model features that describe the sensory (or cognitive) stimuli [1–5]. By doing so, computational neuroimaging methods have been proposed as a means to test the (brain) validity of the algorithm being evaluated and eventually its refinement.

At the single voxel level, two different approaches, population Receptive Fields (pRF) modelling [3] and linearized encoding models [6,7], have been developed to link computational models and fMRI responses. In the following, we will refer to both these approaches indiscriminately as encoding models (see e.g. [8] for the relation between linearized encoding models and pRF approaches).

The performance of a computational model that describes fMRI responses is evaluated in terms of its accuracy in predicting new (test) data. The prediction accuracy is not only affected by inaccuracies in the definition of the algorithm (i.e. mismodelling) but also by other sources of variance in the brain responses that are not expressly modelled (e.g. attention and adaptation) and, most importantly, by physiological (e.g. respiration) and measurement noise. These effects are evidenced by the fact that in real data, presenting multiple times the same stimulus does not result in the same measured brain response. Commonly tested models of sensory (or cognitive) stimuli do not account for the variability in the response between repetitions of the same stimulus which imposes a bound to the ability to encode computational models in fMRI responses. This bound can be interpreted as the performance of the computational model underlying the generation of the responses (i.e. the true underlying model) conditional to the noise (experimental, physiological or other) that is present in the test data (under the assumption of infinite training data). It should be noted here that this represents one of many possible definitions of a bound to the performance of a computational model. This bound is imposed exclusively by the measurement noise in the test data (i.e. *test-data-noise ceiling*). A more realistic definition of the noise ceiling would also consider the influence of the size of the training set and the algorithms used for estimating the computational model, however it has not been proposed yet. Reporting the *test-data-noise ceiling* allows assessing the quality of the predictions obtained when using computational modelling approaches relative to the quality of the data, and thus comparing modelling efforts on different datasets across labs.

In the neuroimaging community, it has been recommended to report the performance of a computational model with respect to the (test data) noise ceiling and, in some cases, these recommendations have led to the use of normalized accuracy scores (e.g. dividing the accuracy by the noise ceiling, [9–13]). Different estimation procedures have been proposed for the *test-data-noise ceiling* but the properties of these different estimators have not been compared. The main purpose of this article is to provide a framework in which the concept of the *test-data-noise ceiling* can be clarified for the users of encoding models and in which different estimators can be compared on the basis of their assumptions.

To illustrate the concept of *test-data-noise ceiling* we can consider the two-step procedure that in many cases is used when fitting a computational model. In the first step, responses to the stimuli are estimated from the whole fMRI time series and, in the second step, the computational model is fit to the stimulus response series. While this two-level procedure is not used by all encoding approaches in practice, the unmodelled variability of the response between repetitions of the same stimulus limits the performance of a computational model that predicts the whole fMRI time series as well (see e.g. pRF models [3]). As a consequence of this hierarchical estimation framework, the prediction accuracy of the model (step 2) is bound by the uncertainty in the estimation of the response (step 1). This bound corresponds to the intraclass correlation coefficient [14] a well-known result in multilevel modelling.

The noise ceiling can be obtained considering the variability across subjects (see e.g. [15]), but here we will focus on estimation procedures at the single subject level, where two approaches have been proposed to estimate the *test-data-noise ceiling* of single voxels. The first models the response of a voxel as a univariate normal distribution with two variance components [16]. The first variance component corresponds to the variability of the signal around its mean due to genuine differences in the brain response between different stimuli (excluding the effects of measurement noise). The second variance component corresponds to the variability in the brain response due to measurement noise. Having an estimate of the measurement noise allows generating new samples for both the signal without noise (genuine brain response) and the measurement (i.e. signal plus noise) using Monte Carlo simulations. The noise ceiling (measured with correlation or predictive R^2^) is then computed using the simulated signals and measurements (i.e. considering the performance in predicting the noisy measurements of a model whose prediction is the clean signal). In what follows we will refer to this approach as the Monte Carlo noise ceiling (MCnc). Alternatively, the noise ceiling can be estimated as the correlation between the estimates of the responses in two independent repetitions of the same experimental procedure [17,18]. In absence of two repetitions of the test set, the split-half noise ceiling estimator (SHnc) can be estimated by splitting the available test data in two disjoint sets (i.e. splitting the trials of all test stimuli in two sets to obtain two estimates of the test data), computing the split-half correlation and applying a correction factor that accounts for the reduced number of trials in each half of the data compared to the full dataset. In this article, we describe the differences between these two noise ceiling estimators using simulated data and derive an analytical solution to the calculation of the *test-data-noise ceiling* obviating the need of computationally demanding procedures (i.e. Monte Carlo simulations) or splitting the data in two sets. Importantly, the MCnc and the analytical noise ceiling we propose are based on an estimate of the variability of the estimated responses due to the measurement noise. In simulations, we show how different estimators for this variability impact the resulting *test-data-noise ceiling* depending on the structure of the noise in the data.

When using linearized encoding approaches, regularization is often required because of the dimensionality of the model with respect to the number of stimuli and because of collinearity between the features of the computational model. Here we evaluate how the bias variance tradeoff introduced by the regularization influences the performance of an ideal model and thus the relationship with the noise ceiling (which is model independent) by imposing a second constraint.

Finally, we evaluate the differences between the noise ceiling estimators using real fMRI data, obtained from 7 Tesla acquisitions. Our results are discussed in terms of their implications for the evaluation of computational models and their comparison using fMRI data.

## Methods

We consider a two-level procedure to fit a computational model to fMRI responses. At the first level, fMRI responses to single stimuli are estimated from the fMRI time series and, at the second level, the computational model is (linearly) fit to the vector of fMRI responses in order to derive the parameter weights (in the space of the features of the computational model).

**Fig 1:**
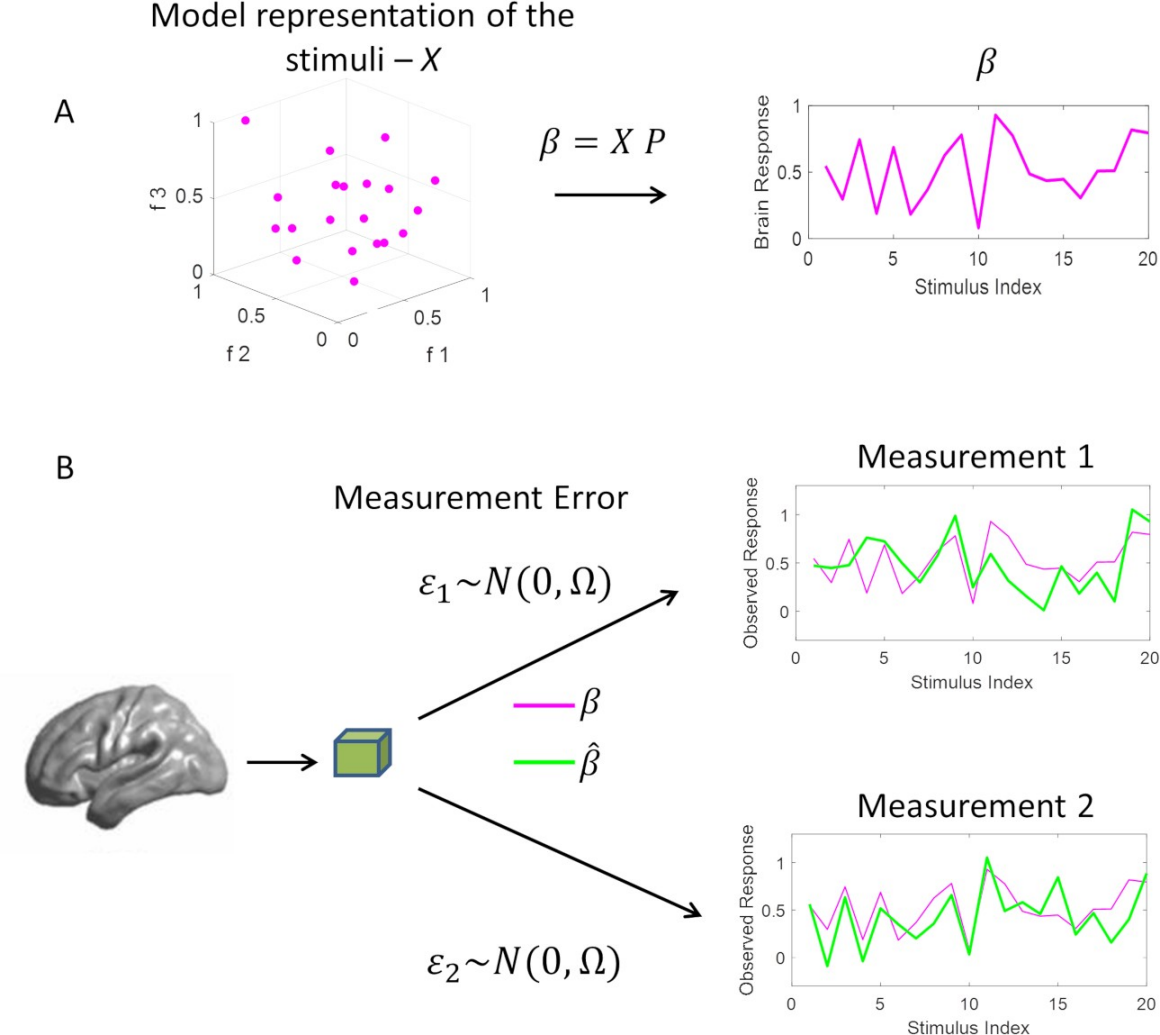
General description of linking a computational model to fMRI brain responses. Encoding approaches assume brain responses (pink in panel A) to be a linear function of the model based representation of the stimuli X (pink dots) (A). Observed fMRI responses (B) are affected by experimental noise which causes estimated responses (green in panel B) to be different from responses predicted by the computational model (pink in panel B) and to be different across repetitions of the same experiment.

Fig 1 illustrates key concepts that underlie the notion of noise ceiling. Brain responses (***β***) can be considered a (linear) function, with weights representing the population receptive field (***P***), of the representation of the stimuli in the space of a computational model (***X*** with 3-features in Fig 1A). The measurement of brain responses (Fig 1B) is affected by experimental/measurement noise such that the estimate of the responses of a given voxel to the same stimuli in two independent measurements (Fig 1B) differs. Fitting a computational model to the measured brain responses allows linking the model features to the measurements and thus predicting voxel responses to new (test) stimuli. When this procedure is used on noisy measurements, the experimental noise imposes a limit to the performance of the model in predicting the measured responses.

In what follows we first describe the two-level fitting approach and the different metrics used to evaluate model fitting in order to introduce some relevant concepts, next we introduce a generative framework and derive the bound to the performance. The mathematical notation used in the following sections is presented in Table 1.

**Table 1:** Mathematical notation.

Symbol	Definitions (size)
***y***	Voxel time series (time points x 1)
**Φ**	fMRI design matrix (time points x num of stimuli)
$\Omega ,\hat{\Omega }$	True and estimated covariance matrix of the fMRI noise (num of time points x num of time points)
$\sigma_{\varepsilon_{t}}^{2}$	Variance of the noise of the fMRI time series for one voxel (scalar)
$\beta ,\hat{\beta }$	True and estimated brain responses for one voxel (num of stimuli x 1)
${\hat{\beta }}_{i}^{r}$	Voxel estimated brain response for the stimulus *i* at fMRI run *r* (scalar)
$V_{\hat{\beta }},{\hat{V}}_{\hat{\beta }},{\hat{V}}_{{\hat{\beta }}_{ii}}$	Voxel true and estimated $\hat{\beta }$ covariance matrix (num of stimuli x num of stimuli); and component *i* from the diagonal of the $\hat{\beta }$ covariance matrix (scalar).
β^*^	Voxel brain responses predicted on the test data with the computational model (num of test stimuli x 1)
*X*,X^*^	Computational model for training and test data respectively (num of training/testing stimuli x num of features)
$P,\hat{P}$	Voxel true and estimated receptive field vector (number of features x 1)
***λ***	Regularization parameter
$\bar{\beta },\bar{\hat{\beta }},\sigma_{\beta }^{2},\sigma_{\hat{\beta }}^{2}$	Voxel mean and variance of the true and estimated voxel brain response across components. (scalars)

### Estimation of the response to single stimuli

The observed fMRI response is assumed to be linearly dependent on the stimuli (design matrix) [19,20] and the estimation (for every voxel) is achieved using generalized least squares (GLS):

$$\hat{\beta }={\left(\Phi^{T}{\hat{\Omega }}^{-1}\Phi \right)}^{-1}\Phi^{T}{\hat{\Omega }}^{-1}y$$ (1)

The covariance matrix for $\hat{\beta }$ is:

$$V_{\hat{\beta }}={\left(\Phi^{T}{\hat{\Omega }}^{-1}\Phi \right)}^{-1}\Phi^{T}\Omega^{-1}\Phi {\left(\Phi^{T}{\hat{\Omega }}^{-1}\Phi \right)}^{-1}$$ (2)

For every voxel, $\hat{\beta }$ is the vector of the estimated responses to the stimuli, ***y*** is vector of the voxel time course (the observed fMRI signal) and **Φ** is the design matrix describing the timing of presentation of the stimuli in the experiment, including the effect of the hemodynamic response. The matrix $\hat{\Omega }$ is the estimated covariance matrix of the fMRI noise, while the **Ω** is the true value of this covariance. This general formulation can accommodate a variety of estimators for computing $V_{\hat{\beta }}$ which depends on the assumptions made with respect to **Ω**. Note that $V_{\hat{\beta }}$ depends of the true **Ω** which is unknown. In practice, estimators of **Ω** should be obtained which leads to the estimator of $V_{\hat{\beta }}$ [21]:

$${\hat{V}}_{\hat{\beta }}={\left(\Phi^{T}{\hat{\Omega }}^{-1}\Phi \right)}^{-1}$$ (3)

The assumption of identically independent distributed (i.i.d) noise (which implies $\hat{\Omega }=I$) leads to the ordinary least squares estimator (OLS) [20]. Violations of the i.i.d. assumptions (i.e. presence of temporal dependences and lack of stationarity of the fMRI noise) involve computing $\hat{\Omega }$ which is an ill conditioned problem, which is usually solved by imposing some form of regularization. Typically, this is achieved by parametrizations of the noise covariance matrix that accommodate the assumptions about the noise structure (e.g. first order autoregressive model)[22–24].

### Linearized encoding models

Linearized encoding models assume the estimated response vector $\hat{\beta }$ to be linearly dependent on the description of the stimuli on the basis of a computational model represented by a matrix ***X*** that projects each of the *n* stimuli onto the model space described by a model with *f* features (Fig 1A). The (linear) weights that link the computational model to the fMRI response are referred to as the population receptive field of the voxel. If the objective of the encoding approach is to model the differences in the brain responses between stimuli as a function of the computational model then, voxels that differ only in their means, but otherwise represent stimuli in the same way, should have the same estimated population receptive field. To do this, the mean of the brain responses (across all stimuli) can be removed from the response vector $\hat{\beta }$ before fitting the model ***X***, or a column of ones can be added to the model ***X*** before fitting the un normalized responses [25]. When there is collinearity across the features or when the number of stimuli *n* is smaller than then number of features *f*, regularization is used. Here we consider the use of ridge regression [26] for estimating the population receptive field $\hat{P}$ linking the computational model (represented by the training data matrix ***X***) to the estimated voxel response vector on the training data set $\hat{\beta }$:

$$\hat{P}={\left(X^{T}X+\lambda I\right)}^{-1}X^{T}\hat{\beta }$$ (4)

where *λ* is the regularization parameter. $\hat{P}$ allows predicting the responses to new (test) stimuli by considering $\beta^{*}=X^{*}\hat{P}$, where X* contains the representation of the stimuli in the test set in the space of *f* features. Other approaches use grid search or more sophisticated optimization algorithms when a non-linear relationship between the features and the response is assumed [3,27].

### Evaluating the performance of computational model encoded in fMRI responses

The performance of a computational model can be assessed on test stimuli using e.g. the sample correlation coefficient between the responses predicted by the computational model β* and the estimated brain responses in the test data $\hat{\beta }$ (see Eq 1):

$$\rho =\frac{\frac{1}{\left(n-1\right)}\sum_{i=1}^{n}\left({\hat{\beta }}_{i}-\bar{\hat{\beta }})(\beta_{i}^{*}-{\bar{\beta }}^{*}\right)}{\sqrt{{\hat{\sigma }}_{\hat{\beta }}^{2}{\hat{\sigma }}_{\beta^{*}}^{2}}}$$ (5)

Where $\beta_{i}^{*}$ and ${\hat{\beta }}_{i}$ are the predicted and estimated response to stimulus *i* respectively. Estimated variances refer to the variability between the components of the vector of responses around the mean of the vector: ${\hat{\sigma }}_{\hat{\beta }}^{2}=\frac{1}{n-1}\sum_{i}^{n}{\left({\hat{\beta }}_{i}-\bar{\hat{\beta }}\right)}^{2}$. The estimated mean $\bar{\hat{\beta }}=\frac{1}{n}\sum_{i}^{n}{\hat{\beta }}_{i}$ corresponds to the sample mean of the estimated response across its components (each component corresponds to one presented stimulus), with consistent definitions for $\sigma_{\beta^{*}}^{2}$ and ${\bar{\beta }}^{*}$. Alternatively, predictive *R*^2^ is frequently used for describing the performance of an encoding model:

$$R^{2}=1-\frac{\sum_{i=1}^{n}{\left({\hat{\beta }}_{i}-\beta_{i}^{*}\right)}^{2}}{\sum_{i=1}^{n}{\left({\hat{\beta }}_{i}-\bar{\hat{\beta }}\right)}^{2}}$$ (6)

Note that we are computing the explained variance between the observed and predicted brain responses $\hat{\beta }$ and β*, which is different than the explained variance at the level of the fMRI time series ***y***. When computed on independent test data, *R*^2^ is defined in the interval [−∞,1]. It is important to note that, while *R*^2^ is sensitive to scaling transformations of the estimated response, the correlation coefficient measures the similarity between the predicted and observed responses in term of covariations around their mean and is insensitive to scaling transformations. This difference between the metrics is relevant when regularization is used. The relation between predictive *R*^2^ and *ρ* (see S1 Text) can be rendered explicit considering that (without loss of generality) the estimated responses were centred to have zero mean ($\bar{\hat{\beta }}$ = 0) [25]:

$$R^{2}=2\rho \gamma (\lambda )-{\gamma (\lambda )}^{2}-\frac{n}{\left(n-1\right)}\frac{{{\bar{\beta }}^{*}}^{2}}{{\hat{\sigma }}_{\hat{\beta }}^{2}}$$ (7)

Where $\gamma^{2}(\lambda )=\frac{{\hat{\sigma }}_{{(\lambda )\beta }^{*}}^{2}}{{\hat{\sigma }}_{\hat{\beta }}^{2}}$ is the ratio between the estimated variances of the predicted and observed response vectors and dependent on the amount of regularization used to estimate the computational model (see Eq 4 in [5]). The ${\bar{\beta }}^{*}$ represents the bias in the mean of the predicted response vector (i.e. how much the mean of the predicted response vector differs from zero, See S1 Text). Note that the optimal *λ* for the maximization of *R*^2^ does not necessarily corresponds to the *λ* which maximizes *ρ*. The predictive squared Euclidean distance *D*^2^ between the vectors $\hat{\beta }$ and β*, which is also a frequently used metric, is closely related to the explained variance: $D^{2}=(n-1){\hat{\sigma }}_{\hat{\beta }}^{2}(1-R^{2})$.

### Estimating the performance of linearized encoding models

As a consequence of the estimation procedure highlighted above (see Eq 1), in linearized encoding models, the estimated response vector $\hat{\beta }$ is assumed to be multivariate normally distributed around the true response vector ***β*** and with covariance matrix that reflects the variability of the $\hat{\beta }$ estimator:

$$\hat{\beta }∼N(\beta ,V_{\hat{\beta }})$$ (8)

For linearized encoding models, ***β*** (i.e. the expected value of the estimated response $\hat{\beta }$) can be considered to be generated on the basis of the computational model defined by the matrix ***X***. In particular we can consider:

β=XP (9)

This assumes a fixed linear relation between matrix of model features ***X*** and the underlying “true” brain response ***β*** (not influenced by measurement noise) which is mediated by the population receptive field vector ***P***. Note that the true response ***β*** can have any shape and is not limited to be a standard gaussian variable. The noise ceiling estimated under such fixed relationship is based on the assumption that the total variance of the underlying ***β*** can be explained by the features contained in ***X***.

### Noise ceiling definition and estimation procedures

For infinite training data (and without the use of regularization) the noise ceiling can be defined as the expected performance (measured as correlation or predictive *R*^*2*^) of the model underlying the generation of the responses (i.e. the “true” model ***X***). Such model uses the true pRF (***P***) and produces correct predictions for a voxel, i.e. it assumes that the predicted responses β* are the true ***β***:

$$\rho_{NC}=E{\left(\rho \right)}_{\beta^{*}|\lambda =0,n_{tr}=\infty }$$ (10)

This definition of the noise ceiling is a function of the variability of the test data, is by construction independent of the computational model, and thus is better referred to as *test-data-noise ceiling*. Different assumptions underlying the univariate modeling of fMRI data result in different estimates of the responses ($\hat{\beta }$) and their variance. Thus, the (true) noise ceiling is affected by the fMRI response estimation procedure. More sophisticated noise ceiling definitions might also consider the performance of the true model conditioned to the actual amount of data available, or conditioned on the particular algorithm that was used to fit the computational model (e.g conditioned on the value of λ).

#### Split-half noise ceiling (SHnc)

The split-half noise ceiling estimator is an empirical procedure which consists of correlating the $\hat{\beta }$ responses to the same stimuli obtained from two disjoint sets of (test) data: (${\hat{\beta }}_{1}$ and ${\hat{\beta }}_{2}$) at every voxel [18, 29]. Because the number of stimuli in each split is reduced by half, the observed correlations are adjusted using the correction:

$$\begin{array}{l} \rho =\frac{\mathrm{cov}\left({\hat{\beta }}_{1},\;{\hat{\beta }}_{2}\right)}{\sqrt{{\hat{\sigma }}_{{\hat{\beta }}_{1}}^{2}{\hat{\sigma }}_{{\hat{\beta }}_{2}}^{2}}}\\ \rho_{\mathrm{SHnc}}=\sqrt{\frac{2\rho }{\rho +1}} \end{array}$$ (11)

The split-half noise ceiling is a non-parametric procedure since it does not rely on the estimation of the $\hat{\beta }$ variances. Therefore, the split-half noise ceiling estimator procedure takes into account all sources of variability that affect the brain responses. Note that the SHnc is defined only for positive split half correlation values. Here we define the SHnc to be zero for negative split half correlations, which is equivalent to assuming that in the case in which the observed correlation between two independent measurements of the same stimuli is negative the maximum performance that any encoding model can achieve is the chance level.

#### Monte Carlo noise ceiling (MCnc)

The MCnc assumes each element of the response vector $\hat{\beta }$ (i.e. ${\hat{\beta }}_{i}$, with *i* running across the *n* stimuli) to be samples of the same univariate normal distribution [16]. The variance of the responses are split in two components, the experimental noise ($\sigma_{\varepsilon }^{2}$) and the variability between stimuli of the *noise free* ***β*** responses ($\sigma_{\beta }^{2}$).

$$\begin{array}{l} {\hat{\beta }}_{i}∼N\left(\bar{\hat{\beta }},{\hat{\sigma }}_{\hat{\beta }}^{2}\right)\\ {\hat{\sigma }}_{\hat{\beta }}^{2}={\hat{\sigma }}_{\varepsilon }^{2}+{\hat{\sigma }}_{\beta }^{2} \end{array}$$ (12)

where ${\hat{\sigma }}_{\varepsilon }^{2}$ is the pooled variability of each component of the response vector due to measurement errors (i.e. ${\hat{\sigma }}_{\varepsilon }^{2}=\frac{1}{n}\sum_{i=1}^{n}{\hat{V}}_{{\hat{\beta }}_{ii}}$). Note that in the definition provided by [16], the experimental noise variability (${\hat{\sigma }}_{\varepsilon }^{2}$) is across experimental conditions (i.e. stimuli) and not across trials (i.e. repetitions) of the same stimulus. When pooling across stimuli the MCnc assumes that the noise affects all stimuli in the same way. An estimate of the variability of the *noise free* signal can be obtained as the difference between the variability of the responses across the *n* stimuli (${\hat{\sigma }}_{\hat{\beta }}^{2}$) and the variability of the noise:

$${\hat{\sigma }}_{\beta }^{2}={\hat{\sigma }}_{\hat{\beta }}^{2}-{\hat{\sigma }}_{\varepsilon }^{2}$$ (13)

After the variability of the *noise free* signal is obtained with Eq 12, Monte Carlo samples of the *noise free* signal can be generated. For every generated noise free signal the experimental noise is added (using a normal distribution with zero mean and variance ${\hat{\sigma }}_{\varepsilon }^{2}$) and the correlation between the *noise free* signal and the *noise contaminated* signal is computed. The median across all simulations represents an estimate of the noise ceiling.

#### Analytical derivation of noise ceiling

Considering the definition provided in Eq 10, if the predicted responses β* are identical to the true expected value of the responses ***β*** the prediction accuracy expressed through the correlation coefficient takes the form of:

$$\rho_{NC}=\frac{\frac{1}{n-1}\sum_{i=1}^{n}\left({\hat{\beta }}_{i}-\bar{\hat{\beta }})(\beta_{i}-\bar{\beta }\right)}{\sqrt{{\hat{\sigma }}_{\hat{\beta }}^{2}{\hat{\sigma }}_{\beta }^{2}}}$$ (14)

Based on the assumption that the measurement error and the true ***β*** are independent, the noise ceiling can be estimated as (see S1 Text):

$${\hat{\rho }}_{NC}=\frac{{\hat{\sigma }}_{\beta }^{2}}{\sqrt{{\hat{\sigma }}_{\hat{\beta }}^{2}{\hat{\sigma }}_{\beta }^{2}}}=\frac{{\hat{\sigma }}_{\beta }}{{\hat{\sigma }}_{\hat{\beta }}}$$ (15)

This means that the noise ceiling can be expressed as the square root of the ratio between the variance of the true brain response (${\hat{\sigma }}_{\beta }^{2}$) and the variance of estimated brain response (${\hat{\sigma }}_{\hat{\beta }}^{2}$), or equivalently the ratio between the variance of the noise free brain response (${\hat{\sigma }}_{\beta }^{2}$) and the variance of the noise contaminated brain response (${\hat{\sigma }}_{\hat{\beta }}^{2}$) (see Eq 12 for the relation between both variances). The denominator ${\hat{\sigma }}_{\hat{\beta }}^{2}$ is easy to compute since $\hat{\beta }$ is estimated from the fMRI time series and its variance can be directly derived from the test data. However, the numerator ${\hat{\sigma }}_{\beta }^{2}$ has to be derived based on the covariance matrix of the estimated $\hat{\beta }$. The analytical noise ceiling can be estimated using the variance estimates of each component of $\hat{\beta }$ according to the formula:

$${\hat{\rho }}_{NC}=\frac{\sqrt{{\hat{\sigma }}_{\hat{\beta }}^{2}-\frac{1}{n}\sum_{i}^{n}{\hat{V}}_{{\hat{\beta }}_{ii}}}}{{\hat{\sigma }}_{\hat{\beta }}}$$ (16)

The term ${\hat{V}}_{{\hat{\beta }}_{ii}}$ refers to the component *i* of the diagonal of the ${\hat{V}}_{\hat{\beta }}$ matrix. As for the MCnc, this general formulation allows considering different estimators for ${\hat{V}}_{\hat{\beta }}$. Note that in Eq 16 a negative number under the square root can be obtained if the average of the variance within components ($\frac{1}{n}\sum_{i}^{n}{\hat{V}}_{{\hat{\beta }}_{ii}}$ is greater that the variance between components (${\hat{\sigma }}_{\hat{\beta }}^{2}$). This can be caused by one or more stimuli (components of $\hat{\beta }$) with a brain response that is equal to zero. In these cases we define the analytical noise ceiling to be zero, which corresponds to assume that the maximum accuracy that encoding models can reach is the chance level. The same indeterminacy of obtaining a negative estimation of the variance can occur for the Monte Carlo NC (Eq 12) and the considerations made for the MCnc and the analytical noise ceiling are consistent with the ones made for the SHnc in the case of negative correlations between the splits.

The noise ceiling estimator ${\hat{\rho }}_{NC}$ has as expected value ρ_NC_, (see S1 Text II). The noise ceiling estimator for the explained variance R^2^ can be derived transforming the noise ceiling for the correlation coefficient to the R^2^ domain with Eq 7. The analytical noise ceiling estimator has the same expected value than the Monte Carlo noise ceiling estimator with the advantage of not requiring a large computational effort.

### Models of the variance of the estimated brain response

As highlighted above, the MCnc and the analytical approach rely on the estimation of the covariance matrix of the estimated regression coefficients ${(\hat{V}}_{\hat{\beta }})$. Different ${\hat{V}}_{\hat{\beta }}$ estimates will therefore result in different expected values for the noise ceiling. In particular, as highlighted in Eq 3, ${\hat{V}}_{\hat{\beta }}$ depends on the assumptions made about the structure of the noise in the fMRI time series (i.e. the structure of $\hat{\Omega }$ in Eq 3). Different analysis software use different covariance constrains for computing $\hat{\Omega }$; to account for autocorrelation in the noise, here we followed the SPM-12 (http://www.fil.ion.ucl.ac.uk/spm/software/spm12) approach which estimates the brain response in two passes. In the first pass, estimated brain responses are computed assuming $\hat{\Omega }=I$, which corresponds to OLS. Next, the noise covariance matrix is computed assuming the same correlation structure for each voxel within each session. A parametrized model of the noise covariance matrix is fit to the pooled covariance matrix using restricted maximum likelihood (spm_reml.m). The covariance constraint for $\hat{\Omega }$ are obtained with the function (spm_Ce.m) with autocorrelation coefficient of 0.2 (empirically determined). In the second pass the $\hat{\beta }$ are obtained with Eq 1 which uses the estimate of $\hat{\Omega }$ derived in the first pass. To account for non-stationarity we followed the RobustWLS toolbox approach (http://www.diedrichsenlab.org/imaging/robustWLS.html). In particular, the estimation of $\hat{\Omega }$ is performed using a Newton-Raphson algorithm (spm_rwls_reml.m in the WLS toolbox) [30]. Note that autocorrelation and the non-stationarity constraints can be combined in the same estimation of $\hat{\Omega }$ [30]. However, we do not perform this combined estimation here since we used WLS as implemented in the RobustWLS toolbox.

When data are acquired across multiple runs (or sessions), additional sources of variability across runs (sessions), can have an effect on the variance of the $\hat{\beta }$ [31]. See [32] for a proper partitioning of the variance of the fMRI into runs and sessions components. Ignoring this source of variability resulted in the underestimation of the variability of the brain responses with the consequence of overestimating the NC. A solution for this problem is to estimate, for each voxel, the $\hat{\beta }$ responses using a mixed effect model. Two reasons limit the use of mixed models in fMRI: first, estimating such model for each voxel has a large computational cost (impractical for a large number of voxels); second, and more relevant, the design may not allow a reliable estimation of the model when the number of presentations of each stimulus across the runs is too small. An alternative is to compute the variability of $\hat{\beta }$ as the variance of the mean of $\hat{\beta }$ across the fMRI runs (sessions):

$${\hat{V}}_{{\hat{\beta }}_{ii}}=\frac{1}{(n_{r}-1)n_{r}}\sum_{r=1}^{n_{r}}{\left({\hat{\beta }}_{i}^{r}-{\bar{\hat{\beta }}}_{i}^{r}\right)}^{2}$$ (17)

where *n*_*r*_ is the number of runs (or sessions), ${\hat{\beta }}_{i}^{r}$ is an estimate of the response for stimuli *i* in the run *r* and ${\bar{\hat{\beta }}}_{i}^{r}$ is the mean of the response for stimulus *i* across the *n*_*r*_ runs. Note that the variance of the mean of $\hat{\beta }$ across *n*_*r*_ runs is the estimator of the variance of $\hat{\beta }$ divided by the number of runs. Since the noise ceiling is only a function of the measurement error of each component of $\hat{\beta }$, (and not of the covariance between components) only the diagonal elements of the matrix ${\hat{V}}_{\hat{\beta }}$ are relevant (See Eq 16). This estimator of the variance does not rely on assumptions on the particular form of $\hat{\Omega }$ made by the parametric estimation procedure and can be derived directly from the run to run variability of the $\hat{\beta }$. Such estimator has the attractive of not depending of the assumptions regarding the structure of the fMRI noise, however in fMRI experiments with only few runs it could make the estimator less robust.

### Data

#### Simulations

To validate the analytical formulation of the noise ceiling and to compare the effect that different estimators of the variance of the responses have on the noise ceiling we performed simulations under different scenarios for the structure of the noise in the fMRI time series. Simulated fMRI time series were obtained starting from a computational model (***X*** representing 168 [126 training and 42 test] stimuli in a space of 128 features) and assuming a linear encoding model where the responses to the stimuli ***β*** are obtained from a linear combination (the population receptive field) of the features of the model (see Eq 4). The number of stimuli (training and test) and the dimensionality of the computational model were selected in correspondence with the experimental design used for the real fMRI data (see next section). The simulated population receptive field vector ***P*** was sampled from the standard normal distribution.

The **β** vector was then combined with a design matrix **Φ** describing the presentation of the stimuli during an fMRI experiment. We used the design matrix of an fMRI experiment in which stimuli where presented following a fast event related design (as commonly used in fMRI encoding approaches—see real data section) in 6 separate runs. Each simulation was replicated 100 times under identical conditions and we report the average value across the simulations and the [5 95] percentiles of the distribution.

We will refer to the different simulation scenarios on the basis of the structure used for the true covariance matrix of the noise **Ω** and to be distinguished from the assumed covariance matrix of the noise used in estimation $\hat{\Omega }$ (see previous section).

1. **i.i.d noise scenario**: we consider **Ω** = ***I*,** i.e. the fMRI time series was generated using independent identical distributed gaussian noise and we assumed the same ***β*** across all runs. We performed simulations in this scenario, as a function of the ratio between the variance of the signal and the variance of the noise (SNR).
2. **autocorrelated noise scenario**: We generated the noise of fMRI time series following a first order autoregressive processes with varying autoregressive coefficient (implemented using the Matlab function arima.m). The generated noise was scaled to have SNR equal to one. The results were displayed as function of the lag-1 autocorrelation of the fMRI noise. Considering that the estimation of the noise covariance matrix requires a subset of voxels (see previous section), we simulated 1000 voxels with the same noise covariance structure in each simulation, but we report the noise ceiling estimation for one of them (i.e. the first voxel).
3. **non-stationary noise scenario**: the fMRI time series were generated using gaussian i.i.d. noise for which a random subset of 5% of the fMRI volumes of the noise signal were scaled with a non-stationarity factor varying in the interval [1, 3] (See the simulations in [30] for a more detailed description). The non-stationarity factor effectively reduces the SNR for a subset of the volumes, for the other set the SNR was fixed to one. Considering that the estimation of the noise covariance matrix requires a subset of voxels (see previous section), we simulated 1000 voxels with the same noise covariance structure in each simulation, but we report the noise ceiling estimation for one of them (i.e. the first voxel).
4. **autocorrelated noise with run to run variability:** we repeated scenario 1 with the inclusion of autocorrelated noise fixing the lag-1 autocorrelation value to 0.25. Here we evaluated the effect on the noise ceiling of the run to run variability in the response vector ***β***. For each run, ***β*** comprised of two additive terms, one common across runs (coming from the model matrix and the population receptive field: ***XP***), and the second variable across runs modeled as standard gaussian distribution with zero mean. The proportion between the two terms was selected such that the average correlation between two halves of the experiments (first and second twelve runs) reached 0.6. We performed different simulations by varying the amount of SNR now defined as the ratio of the variance of the term of the response common across runs and the variance of the noise.

In each of the simulated scenarios we computed the analytical noise ceiling (Eq 16) using different estimators for $\hat{\beta }$ which are conditioned to the parametric form of $\hat{\Omega }$ that determines ${\hat{V}}_{\hat{\beta }}$. In particular, we tested three estimators depending on different parametric assumptions: 1) $\hat{\Omega }=I$, which corresponds to ordinary least square (OLS), 2) an autoregressive model of order 1 (as implemented in SPM 12), and 3) a parametric model of non-stationarity (using the RobustWLS toolbox). We will refer to these three estimators as $\hat{\Omega }=I$, AR(1) and NST respectively and thus we will refer to the noise ceiling estimates resulting from them using the same acronyms. The noise ceiling obtained with these parametric estimators of the variance of the $\hat{\beta }$ was compared to the noise ceiling obtained using the run to run variability (R2R) for estimating the variance of the response (using the variance of the estimated responses introduced in Eq 17 in the noise ceiling estimate proposed in Eq 16) and the SHnc. Note that for both the R2R noise ceiling and the SHnc the estimated responses $\hat{\beta }$ are obtained using ordinary least squares (i.e. assuming $\hat{\Omega }=I$). This is to validate that the SHnc and the R2R noise ceiling estimator are robust to violations of the OLS assumptions.

In each simulation scenario all noise ceiling estimators were compared to the true noise ceiling that in simulations can be computed directly knowing the true simulated ***β*** using Eq 14. Note that the true noise ceiling is dependent on the estimation procedure used to obtain the test $\hat{\beta }$ (see section Noise ceiling definition and estimation procedures.). As a consequence, the ground truth noise ceiling values used for comparative purposes in the simulations will also differ depending on the estimation used to obtain $\hat{\beta }$.

#### MRI data

The data presented here are part of a larger study that includes ten healthy participants. The subjects had no history of neurological disease, and gave informed consent before commencement of the measurements. The Ethical Committee of the Faculty for Psychology and Neuroscience at Maastricht University granted approval for the study. Magnetic resonance imaging data were acquired on an actively shielded MAGNETOM 7T whole body system driven by a Siemens console at Scannexus https://scannexus.nl/. A Nova Medical head RF coil (single transmit, 32 receive channels) was used to acquire anatomical (T_1_, Proton Density [PD] weighted) and functional (T_2_* weighted BOLD) images. T_1_ weighted (0.7 mm isotropic) images were acquired using an MPRAGE sequence (repetition time [TR] = 3100 ms; time to inversion [TI] = 1500 ms; time echo [TE] = 3.5 ms; flip angle = 5°). PD images were acquired with the same MPRAGE as the T_1_ weighted image but without the inversion pulse (TR = 2160 ms; TE = 3.5 ms; flip angle = 5°), and were used to minimize inhomogeneities in T_1_ weighted images [33]. Acquisition time for the T_1_ and PD datasets were ~ 9 and 4 minutes respectively. Anatomical data were analyzed with BrainVoyager QX and were resampled (with sinc interpolation) in the normalized Talairach space (Talairach and Tournoux, 1988) at a resolution of 0.5 mm isotropic.

Functional (T_2_* weighted) data were acquired using a clustered Echo Planar Imaging (EPI) technique (1.1 mm isotropic; TR = 2.6 s; GRAPPA = 3; MultiBand = 2; Gap = 1.4 s). The experiments were designed according to a fast event-related scheme and slices were prescribed in a coronal oblique orientation in order to cover the brainstem and auditory cortex (Heschl’s gyrus, planum temporale and planum polare) bilaterally. A total of 168 sounds were presented six times across 24 runs in silent gaps in between volme acquisitions using magnetic compatible earbuds (Sensimetrics inc.). The sounds were divided into four training and testing sets (126 and 42 sounds respectively). The four training and test sets (i.e. the four cross validations) where built such that training sounds and test sounds where presented in separate runs (e.g. for cross validation number 1 runs [4, 5, 12, 16, 17, 24] where test runs and the remaining runs where training runs). Within each run, sounds were randomly spaced at a jittered interstimulus interval of 2, 3, or 4 TRs and presented in the middle of the silent gap between acquisitions (leaving 100 ms of silence before and after the sound). Zero trials (trials where no sound was presented [5% of the trials]), and target trials (trials in which a sound was presented two times in a row [5% of the trials]) were included. Subjects were instructed to perform a one-back task, and were required to respond with a button press when the same sound was presented two times consecutively. Target trials were excluded from the analysis. Before starting the experiment (with the ear buds in place), the subjects were instructed to adjust the overall sound intensity to a clearly audible and comfortable level. This resulted in an approximate sound intensity of 65 dB. The total scanning was divided over two sessions that were acquired in two consecutive days.

#### Functional data analysis

Functional data were analyzed with BrainVoyager QX (v20.4) [34]. Preprocessing consisted of slice scan-time correction (with sinc interpolation), 3-dimensional motion correction, and temporal high pass filtering (removing drifts of 4 cycles or less per run). Functional data were co-registered to the anatomical data, normalized in Talairach space (Talairach and Tournoux, 1988), and resampled (with sinc interpolation) at a resolution of 1 mm isotropic.

We calculated the fMRI response to each sound using the following steps. First, for each cross validation, we obtained noise regressors using GLM denoise ([16], http://kendrickkay.net/GLMdenoise/). GLM denoise was run for each cross validation solely on the training runs with a fixed hemodynamic (canonical form). The selected principal components were then used to derive noise regressors in the test data. In the supplementary figures we reported the difference between the NC estimated with and without the use of GLM denoise. Second, the responses to the stimuli were estimated with SPM12 or RobustWLS, also using the canonical hemodynamic function. Similarly to the simulations, in one subject, we report results obtained using different assumptions on the structure of the noise covariance matrix. In particular, we computed $\hat{\beta }$ and ${\hat{V}}_{\hat{\beta }}$ necessary to calculate the analytical noise ceiling (and the MCnc) using: 1) $\hat{\Omega }=I$, which corresponds with OLS estimation, 2) an autoregressive model of order 1 (as implemented in SPM 12), and 3) a parametric model of non-stationarity (using the RobustWLS toolbox). We will refer to these three estimators as: $\hat{\Omega }=I,\;{\hat{\Omega }}_{AR(1)}$, and ${\hat{\Omega }}_{NST}$ respectively. The noise ceiling estimators presented in simulations were compared in one arbitrarily selected subject (using 50000 voxels randomly selected). For this subject the voxels time series, the fMRI design matrix, the $\hat{\beta }$ images (assuming $\hat{\Omega }=I$), together with the Matlab codes for computing the noise ceiling are publically available at: https://zenodo.org/deposit/1489531. In all other subjects, we report the values of SHnc and R2R noise ceiling (50000 voxels randomly selected) obtained using OLS for computing $\hat{\beta }$.

## Results

### Validating the analytical noise ceiling estimator

**Fig 2:**
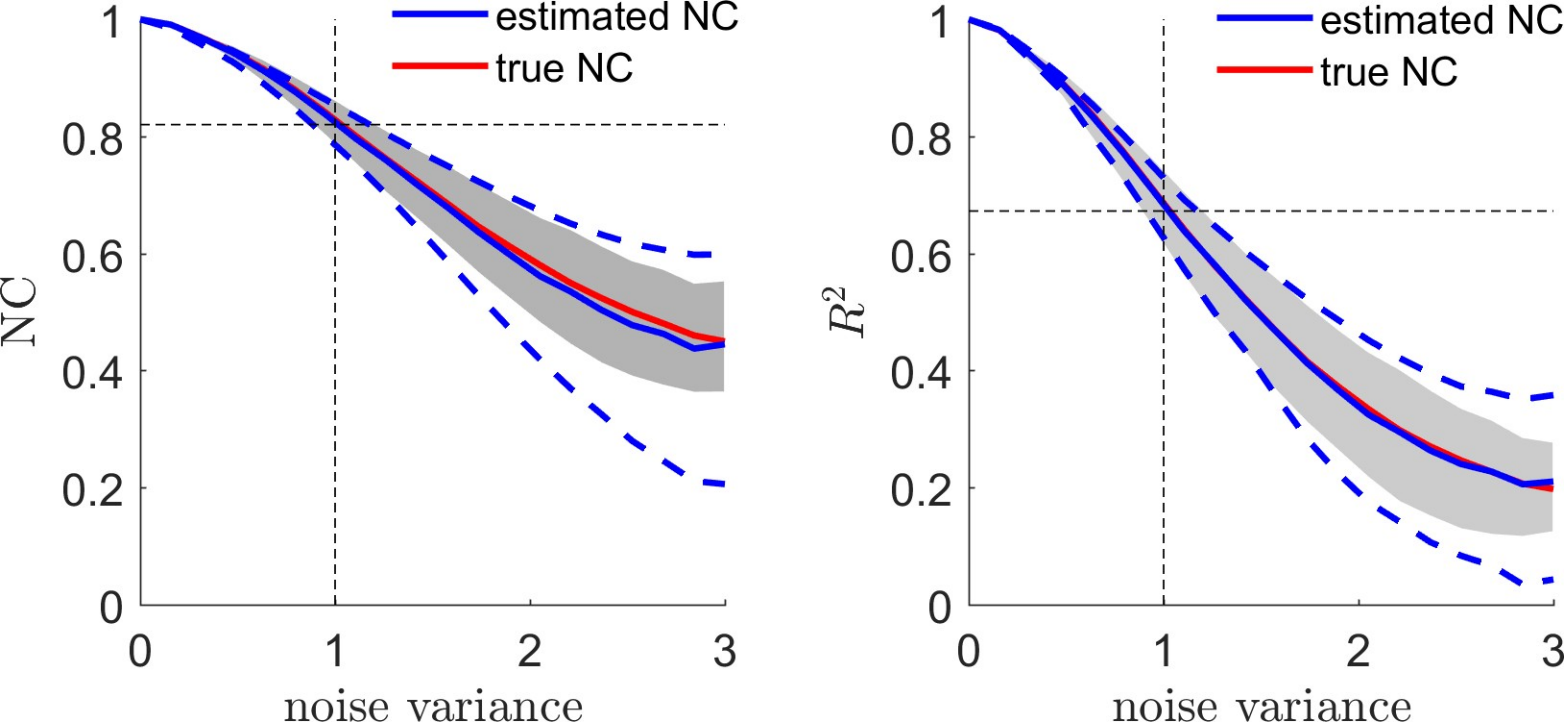
The analytical noise ceiling estimator (blue) and its true value (red) are presented for different levels of variance of the noise (the variance of the signal is equal to one). The left panel shows the noise ceiling for the correlation coefficient while the right panel shows the noise ceiling for the explained variance. The grey shadowed area denotes the [5 95] percentiles of the true NC distribution, while the blue dashed line denotes the [5 95] percentiles for the estimated NC across 100 simulations.

In the i.i.d noise scenario, and using an estimation based on the assumption of noise covariance matrix is equal to the identity matrix, we verified that the expected value of the analytical NC corresponds to its definition of Eq 14 by comparing (mean and [5 95] percentiles across 100 simulations) the estimated noise ceiling value (using Eq 16) with the true noise ceiling that in simulations can be computed directly knowing the true simulated ***β*** using Eq 14 (red line in Fig 2). This comparison is reported in Fig 2 for both correlation and predictive R^2^. The mean value of the analytical NC estimator (blue in Fig 2) matched the mean value and variance of the true noise ceiling. The R^2^ showed stronger dependence with the experimental noise than the correlation coefficient. The noise ceiling estimator showed larger variability (blue dashed line) than the true noise ceiling (shaded region) due to the uncertainty associated with the estimation of $V_{\hat{\beta }}$ (see Eq 3).

### Comparison with the MCnc in the i.i.d scenario

**Fig 3:**
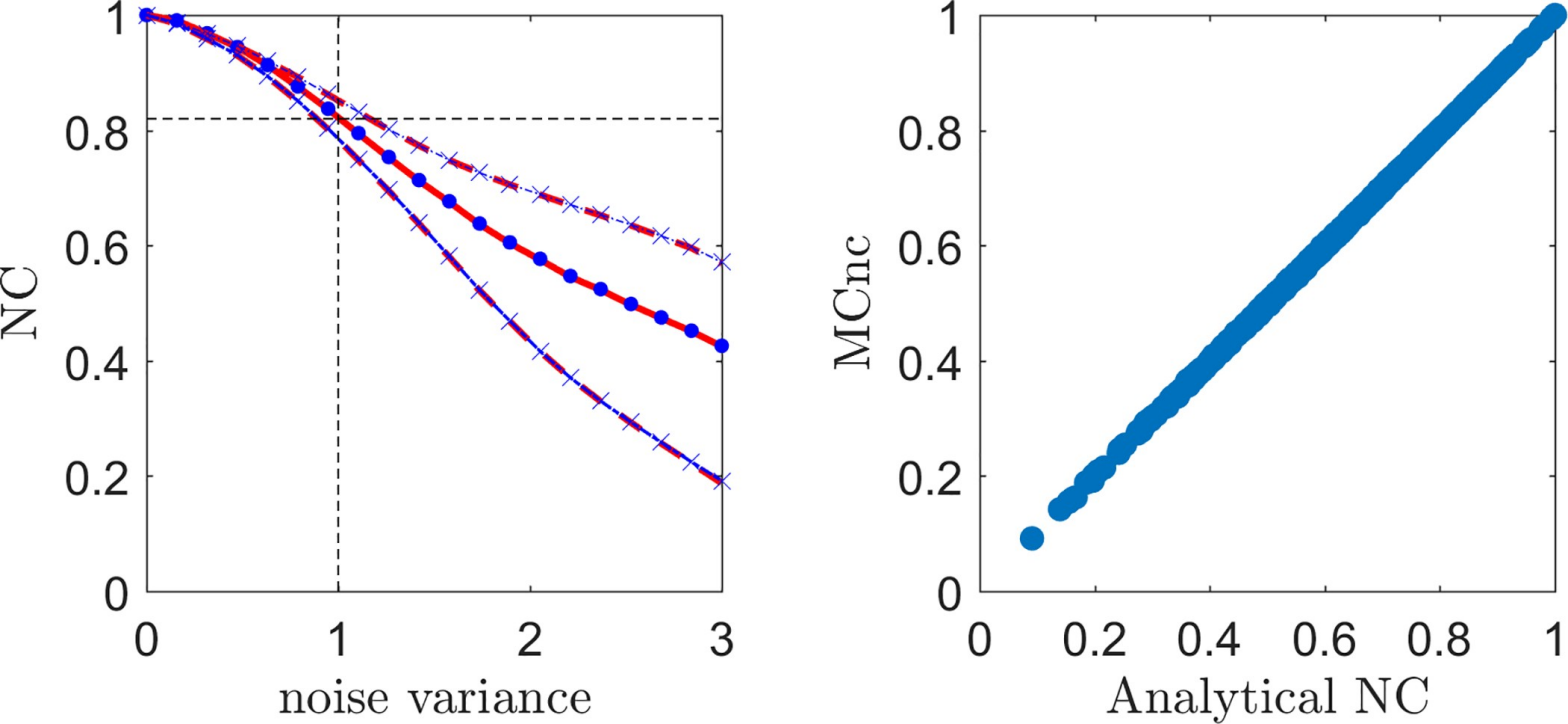
Left Panel: The mean of the NC across the 100 simulations is presented in the red line and blue dots for the Monte Carlo NC (MCnc) and the analytical NC respectively as a function of the experimental noise. Both methods produced identical results thus the curves are perfectly superimposed. The corresponding red dashed lines and blue crosses denote the [5 95] percentiles of the distribution across the 100 simulations. Right Panel: scatter plot of the Monte Carlo noise estimates and analytical noise ceiling estimates.

Using simulated data under i.i.d scenario and estimating ${\hat{V}}_{\hat{\beta }}$ assuming that the noise covariance matrix is equal to the identity matrix we validated the equivalence between the Monte Carlo estimator (MCnc) [16] and the analytical estimator (Eq 16). The results are reported in Fig 3 for the correlation as evaluation metric (for simplicity). Both NC estimators resulted in the same mean and variance across the 100 simulations (the mean and the [5 95] confidence bands are superimposed in Fig 3 left panel). For both NC methods, the variability of the estimated NC increased with decreasing mean value (mean and variance are not independent). The relation between the two NC estimators is presented as a scatter plot in the right panel of Fig 3 for all levels of experimental noise (without averaging across simulations).

The advantage of using the analytical noise ceiling estimator is that it did not require a computationally expensive resampling procedure at each voxel. The difference in computation time was: analytical NC (7x10^-5^ seconds/voxel) vs MCnc (0.06 seconds/voxel, for 1000 Monte-Carlo samples), on a PC with intel(R), i7-6700HQ, CPU 2.6 GHz, 16Gb of RAM processor). We have verified equivalence between the MCnc and the analytical NC also in the other simulation scenarios and using different assumptions about the structure of the covariance matrix of the noise during estimation. As both methods proved to be equivalent we report only the analytical NC estimator for the subsequent analyses.

### Comparison between NC estimators in the i.i.d noise scenario

**Fig 4:**
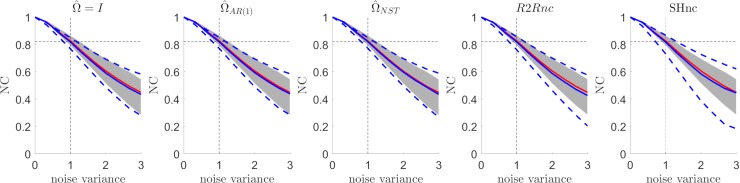
Estimated NC for the different methods for variable levels of noise in the i.i.d scenario. The mean value and the [5 95] percentiles are displayed in continuous and dotted blue lines respectively for the each method. The mean value of the NC computed with the parameters used for generating the data are displayed in red (true NC) with the [5 95] confidence intervals denoted by the shaded region.

Fig 4 summarizes the results (for different simulated noise levels) obtained in estimating the noise ceiling in the i.i.d. noise scenario. The first three panels from the left represent the analytical NC obtained using three different parametric estimates of ${\hat{V}}_{\hat{\beta }}$ (i.e. assuming different structures for the noise covariance matrix during estimation, see Methods section). The analytical NC based on parametric estimators of ${\hat{V}}_{\hat{\beta }}$ are compared to the noise ceiling using the run to run variability for estimating the diagonal entries of ${\hat{V}}_{\hat{\beta }}$ (referred here as R2Rnc) and the split-half noise ceiling estimator (SHnc). In each panel, the true value of the noise ceiling (mean across 100 simulations–red in Fig 4; shaded gray area represents the [5 95] percentile of the true value across the simulations) is compared to the estimated mean (blue in Fig 4 –dashed blue lines represent the [5 95] percentiles). All estimators showed the same mean across simulations (blue line). However the parametric noise ceiling estimators showed less variability (dashed lines) than the non parametric noise ceiling estimator (SHnc and R2R). Between the non parametric noise ceiling estimators the SHnc showed larger variability than the R2Rnc. This simulation proves that all the NC estimators are equivalent when the noise is i.i.d.

### Comparison between estimators in the presence of autocorrelated noise

**Fig 5:**
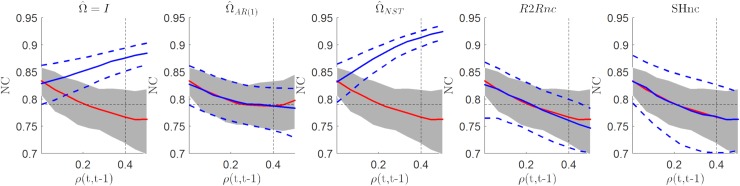
NC estimates using different methods as a function of the lag-1 autocorrelation of the noise. The mean value and the [5 95] percentiles are displayed in continuous and dashed blue lines respectively. The true noise ceiling is displayed in red with the [5 95] confidence intervals denoted by the shaded region.

Fig 5 summarizes the results obtained in estimating the noise ceiling in the case of autocorrelated noise. Each panel reports the result obtained with a different estimation procedure (parametric estimators–first three panels from the left; R2R noise ceiling and SHnc) for different levels of the lag-1 autocorrelation in the noise. The true noise ceiling is also reported (red–mean value across 100 simulations; gray shaded areas [5 95] percentiles). The analytical noise ceiling assuming i.i.d noise ($\hat{\Omega }=I$ - first panel in Fig 5) or using a parametric model of the non-stationarity of the noise (${\hat{\Omega }}_{NST}$, third panel in Fig 5) result in overestimating the NC (with larger overestimation at higher levels of autocorrelation in the noise). The reason for this overestimation is the underestimation of the variance of the brain response (${\hat{V}}_{\hat{\beta }}$). Note that for ${\hat{\Omega }}_{NST}$ we consider only non-stationarity of the noise, but in principle non-stationarity and autoregressive assumptions can be combined in one estimation [30]. In this combined NST-AR(1) estimation we would expect the estimated noise ceiling to approach the true noise ceiling. All other estimators resulted in unbiased (i.e. estimated noise ceiling equal to the true noise ceiling) NC values. Interestingly, violating the noise assumption (e.g. assuming i.i.d. noise instead of autocorrelated noise) also results in a lower true noise ceiling. This effect is visible when comparing the true noise ceiling (red curves) across the panels in Fig 5, and is caused by the increased variance in the $\hat{\beta }$ obtained with e.g. an OLS model compared to estimates obtained with AR(1).

### Comparison between NC estimators in the presence of non-stationary noise

**Fig 6:**
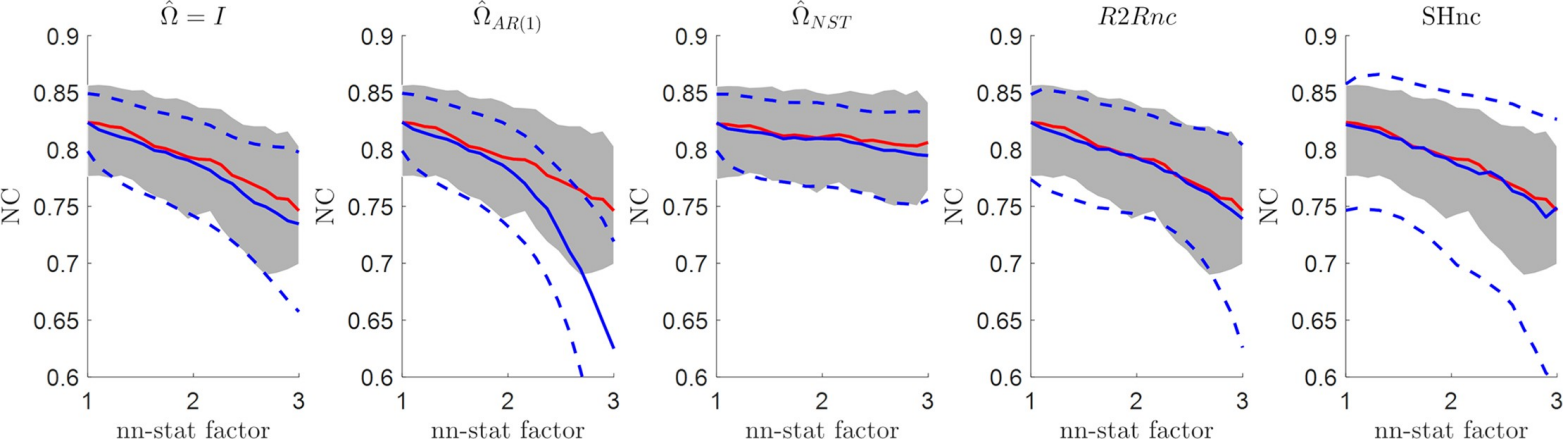
Estimated NC for the different methods as a function of the non-stationarity factor on the noise for a fixed value of the SNR (SNR = 1). The mean value and the [5 95] percentiles are displayed in continuous and dashed blue lines respectively for each method. The mean value of the true noise ceiling is displayed in red with the [5 95] confidence intervals denoted by the shaded region.

The performance of different estimators of the noise ceiling under violations of the non-stationarity assumption for the noise is presented in Fig 6 for an SNR level of one (i.e. when the non-stationarity factor [x-axis in the panels of Fig 6] is equal to one, the simulation is identical to an i.i.d. scenario with SNR = 1). Interestingly, the non-stationarity affected both the true noise ceiling (red curve and shaded grey area in Fig 5) and the estimated noise ceiling (blue curve and dashed blue curves in Fig 5), that are both underestimated when i.i.d. or AR(1) assumptions are used. The SHnc showed the larger variability across simulations. This results from the increased variance for the $\hat{\beta }$ compared to estimates obtained with weighted least squares ${\hat{\Omega }}_{NST}$ [30].

### Comparison between NC estimators in the presence of autocorrelated noise and random effects

**Fig 7:**
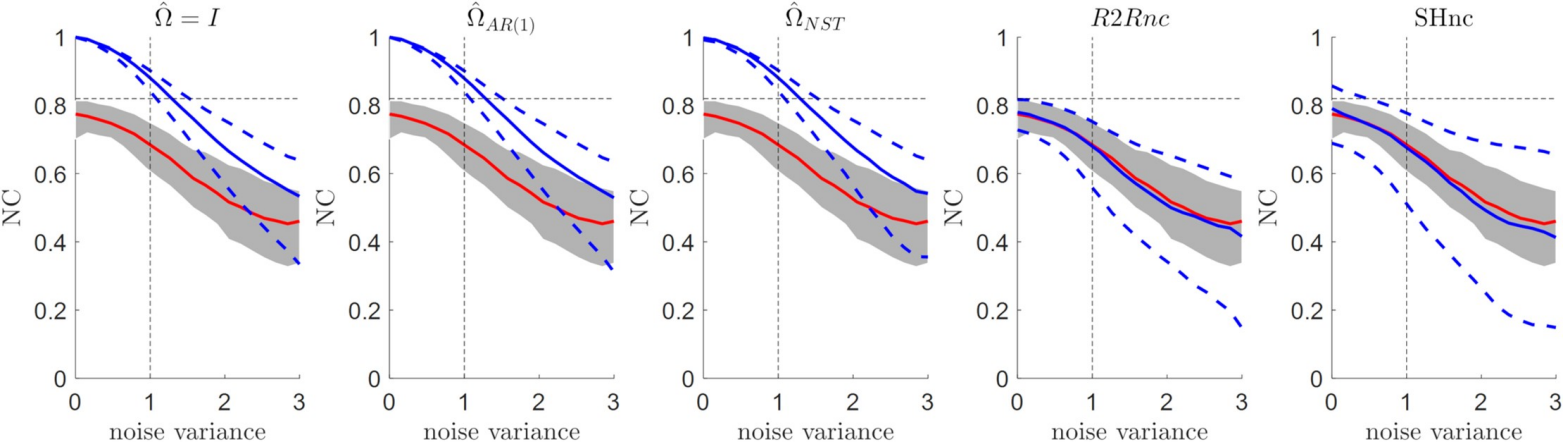
Estimated NC in the presence of autocorrelated noise and run to run variability in the simulated responses as a function of the variance of the noise in the data. The mean value and the [5 95] percentiles are displayed in continuous and dashed blue lines respectively for the each method. The mean value of the true NC is displayed in red with the [5 95] confidence intervals denoted by the shaded region.

The last set of simulations tested the presence of autocorrelation combined with run to run variability in the true ***β*** responses. Fig 7 reports results for the different noise ceiling estimation methods at different levels of variance of the noise (i.e. decreasing SNR) and for a fixed level of autocorrelation in the noise (0.25) which corresponds to the level of autocorrelation observed in the real fMRI data presented in this article (see below). In this scenario only the analytical noise ceiling based on the run to run variability (R2Rnc) and the split-half noise ceiling (SHnc) showed mean values consistent with the true noise ceiling (red curves). The SHnc noise ceiling showed the larger variability across all the noise ceiling methods.

### Influence of sample size and the interaction with the regularization parameter

**Fig 8:**
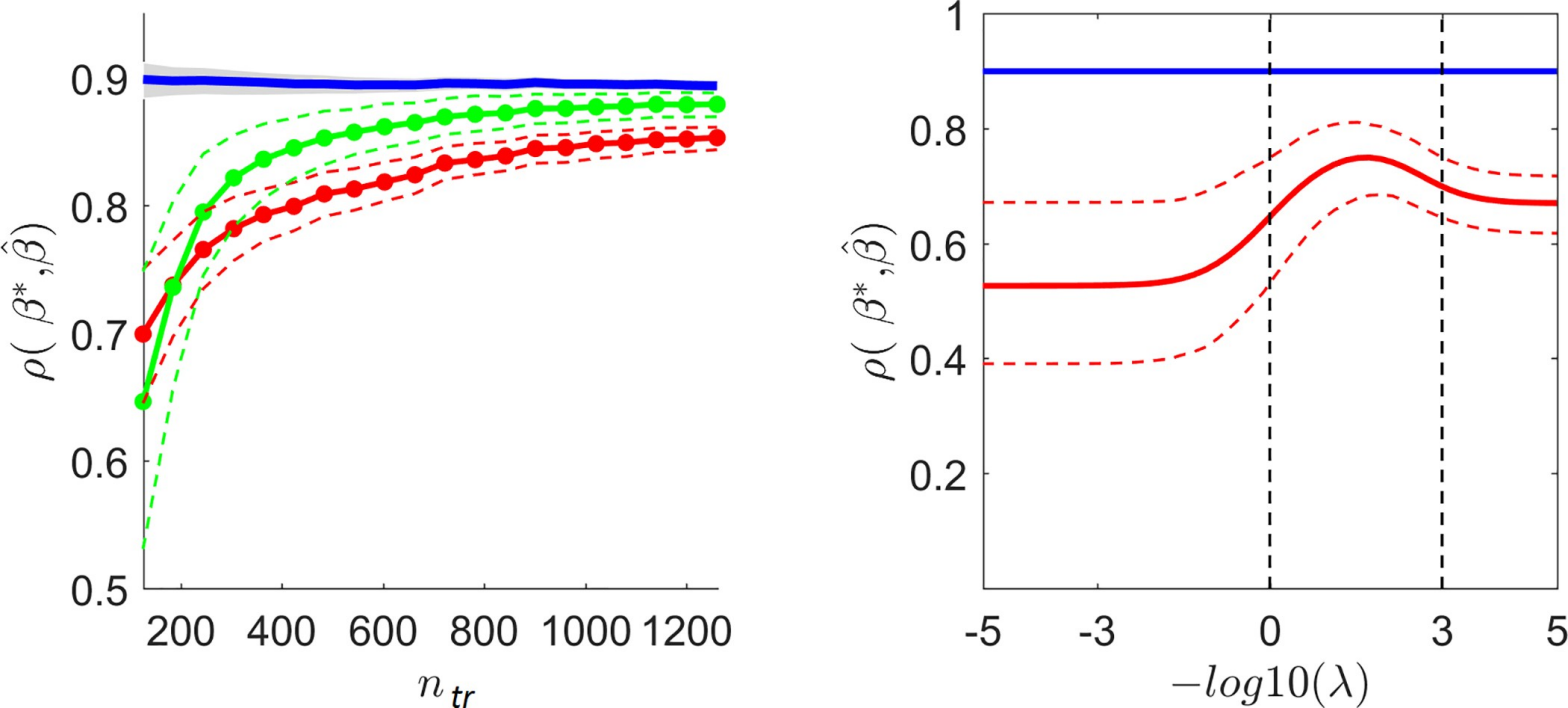
Left panel: Model performance as a function of the number of trials for a fixed level of experimental noise. The analytical noise ceiling estimator for the correlation coefficient in blue and its [5 95] percentiles denoted by the shadowed area is plotted together with the performance of two models that differ in the amount of regularization (green λ = 100; red λ = 103). The corresponding [5 95] percentiles are presented in green and red dotted lines. Right panel: The effect of regularization on the performance. The correlation coefficient is used the metric for describing the performance. The blue line depicts the analytical noise ceiling estimator which is independent of *λ*. The mean accuracy of the model performance and the [5 95] percentiles of the distribution across 100 repetitions of the same simulation are presented by the red dotted line. The vertical bars highlight the λ = 1; red λ = 1000 for correspondence with the left panel.

The left panel of Fig 8 shows, on data simulated with i.i.d. noise, the performance of an encoding model trained using the true underlying computational model (i.e. an encoding model trained using the matrix ***X*** used for simulating the responses). The performance is measured as the correlation between predicted β* and the estimated $\hat{\beta }$ vectors of the test data, for different sample sizes in training (from *n*_*tr*_ = 126 up to 10 times more the number of features *n*_*tr*_ = 1260) and two levels of regularization (λ = 10^0^ and λ = 10^3^) (green and red curves in Fig 8). The variance of the noise was fixed to 0.5.

As expected, prediction accuracy increased asymptotically with sample size. However, as a consequence of the bias-variance trade-off introduced by the regularization procedure, the performance did not reach the noise ceiling even for a very large number of samples. High regularization implies less variability in the estimated linearized model weights (i.e. the pRF in fMRI encoding approaches) as depicted by the narrower [5 95] variability bands. Note that the reduced variability in data poor scenarios comes at the cost of an increased bias highlighted by the larger distance to the noise ceiling in data rich scenarios.

The right panel of Fig 8 depicts in more detail the influence of the regularization parameter. By keeping the experimental noise variance and sample size constant (0.5 and *n*_*tr*_ = 126 respectively) we evaluated the prediction accuracy (measured as correlation) of the model underlying the generation of the data and considering the effect of regularization on the difference between the actual model performance and the noise ceiling. The performance did not reach the noise ceiling even when the optimal regularization parameter was selected (the one that results in the highest performance).

### Noise ceiling in a real data example

**Fig 9:**
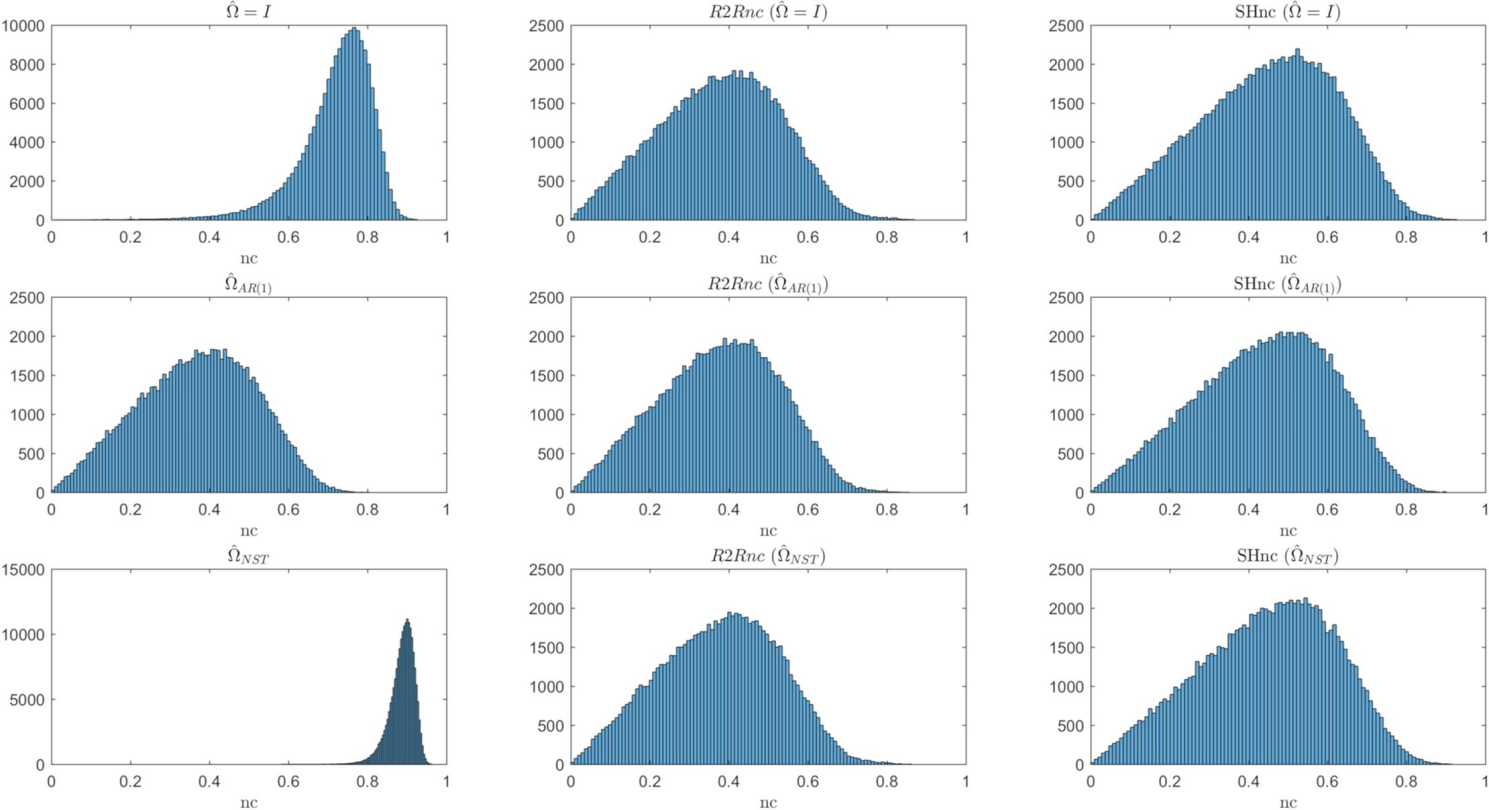
Noise ceiling estimates obtained in real fMRI data. Histograms represent the noise ceiling obtained for 50000 voxels of the temporal lobe for one subject. Every row in the figure presents the noise ceiling estimated under different parametrizations of the noise covariance matrix (from top to bottom: $\hat{\Omega }=I,\;{\hat{\Omega }}_{AR(1)}$ and ${\hat{\Omega }}_{NST}$). The left most columns show the analytical noise ceiling using the estimate of the covariance of the responses based on the different parametric models. The R2Rnc and the SHnc are presented in the two right most columns and, in each row, are computed based on the $\hat{\beta }$ obtained under different parametrizations of the noise covariance matrix.

Using a real fMRI data set we tested the analytical noise ceiling (using parametric and non-parametric estimates for ${\hat{V}}_{\hat{\beta }}$) as well as the SHnc. Fig 9 shows the results obtained with all the tested approaches in one single individual (50000 randomly selected). The column on the left in Fig 9 reports the results for the analytical estimator of the noise ceiling using different estimation of $\hat{\beta }$ and ${\hat{V}}_{\hat{\beta }}$. Similarly to simulations with autocorrelated noise, the results indicate that assuming the noise structure to be either i.i.d or non-stationary (without autocorrelation) results in a higher estimate of the noise ceiling compared to the estimation obtained using an AR(1) assumption (See Fig 5). The second and third column from the left in Fig 9 show estimate of the R2Rnc (analytical NC using run to run variances) and SHnc obtained using different estimates for the response vector $\hat{\beta }$ (i.e. assuming $\hat{\Omega }=I,\;{\hat{\Omega }}_{AR(1)}$ and ${\hat{\Omega }}_{NST}$ from top to bottom respectively). While small differences between the rows can be observed, the R2R noise ceiling and the SHnc are less affected by the method used for the estimation of the response vector compared to the analytical solution that uses a parametric model of the ${\hat{V}}_{\hat{\beta }}$ (left most column in Fig 9).

**Fig 10:**
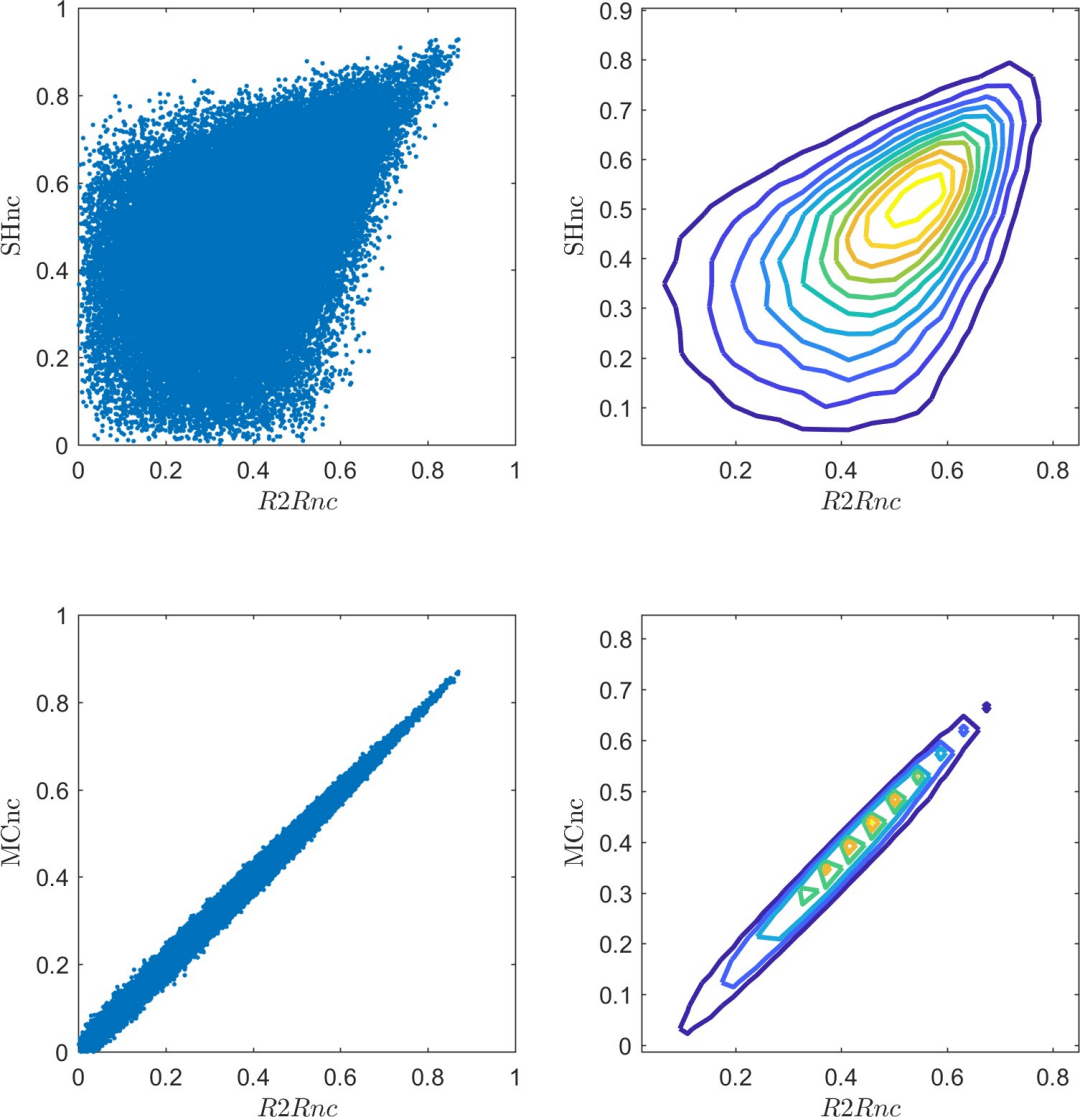
Scatter plot and contour plot of the bivariate histograms of the relationship between: The R2Rnc and the SHnc (upper panel) and the R2Rnc and MCnc (lower panel) for 50000 randomly selected voxels at one subject.

The relationship between the SHnc and the R2Rnc (analytical NC using run to run variances) is presented at the upper panel of Fig 10 for the data of one participant (all the other participants in the dataset showed a similar behaviour). The two NC estimators converged to similar values for high signal to noise ratio (SNR; i.e. when the estimated NC is high). This is in line with what we observed in the simulations where the variance of the estimated noise ceiling was low when the SNR was high. In those voxels where the SNR is low, the variability of the estimators results in a low correlation between the two NC methods. The lower panel of Fig 10, confirmed the equivalence, in real data between the analytical NC of the noise ceiling and the MCnc.

**Fig 11:**
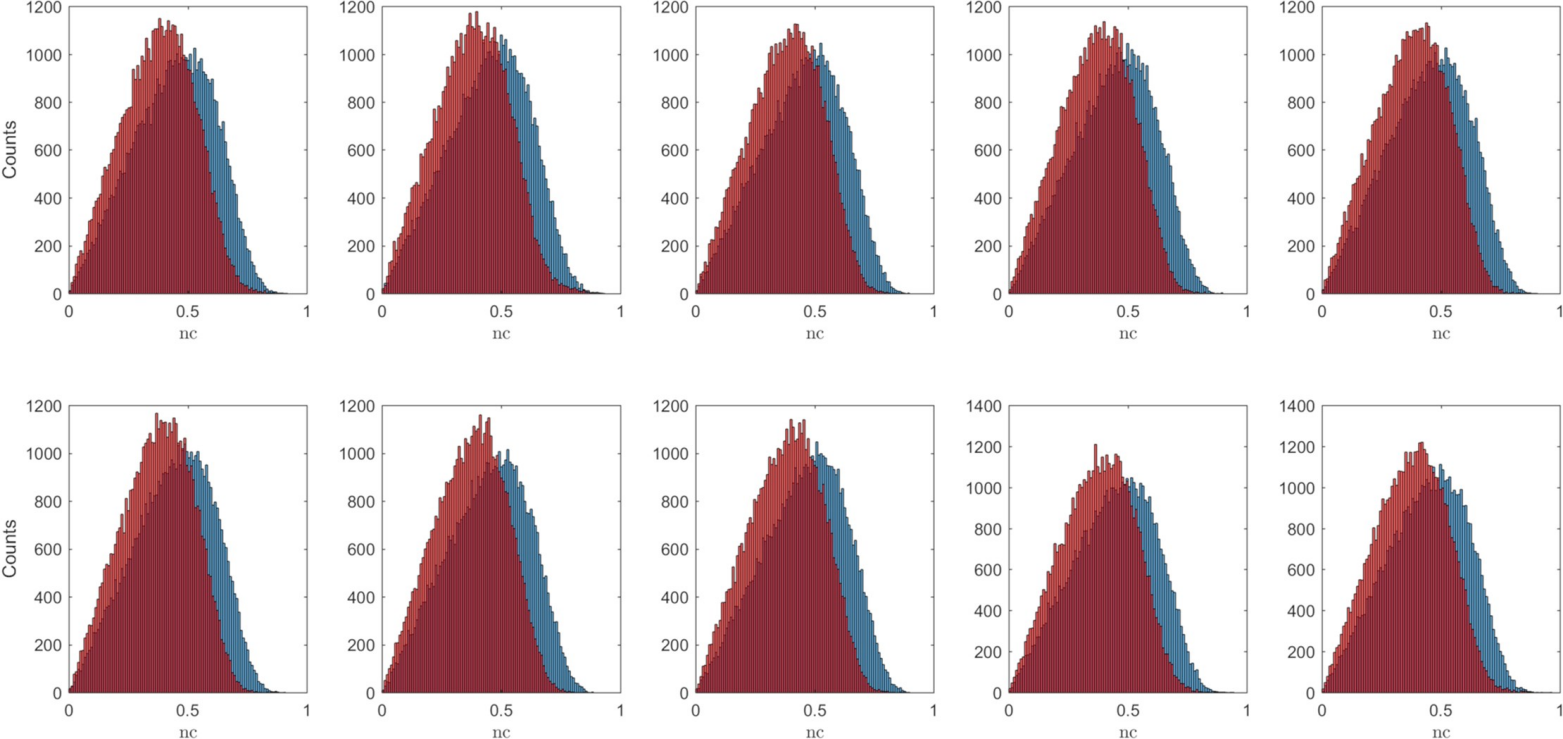
Histograms of the noise ceiling obtained with the split half method (blue) and the analytical NC (with variance the run to run variance R2Rnc; red) across 50000 (randomly selected) voxels for ten subjects.

Fig 11 shows the results obtained for the R2Rnc (red) and the SHnc (blue) in ten subjects (first two rows and the two left most panels in the bottom row). These results are obtained on 50000 voxels (randomly selected) and assuming i.i.d. noise for estimating the voxel responses. The SHnc showed slightly higher mean values compared with the R2Rnc. These results were obtained including the noise regressors in the fMRI design matrix for estimation of the ***β*** (see section Functional data analysis). Removing the noise regressors from the estimation of the responses did not affect the results (see supplementary figures).

## Discussion

In neuroscience applications, computational models are a formal expression of the algorithms that underlie cognitive and sensory processes. Linking a model with non-invasive measures of brain activity (such as fMRI) [35,36] allows testing the accuracy of the algorithm and eventually refine it to produce brain inspired models. Ultimately this approach can produce an understanding of brain function at the level of fundamental processing units. The accuracy of a computational model is measured in terms of its ability to predict the brain responses to sensory (or cognitive) stimuli. In this regard, the intrinsic noise of the fMRI measurements procedure provides a bound to the accuracy of any model and this bound can be referred to as the *test-data-noise ceiling*.

In this article, we evaluated two existing approaches for the calculation of the noise ceiling using simulations and real data. We also proved that the noise ceiling can be computed directly from the variance of the estimated brain responses. In simulations and real data, we demonstrated that the previously introduced MCnc estimator [16] and the analytical NC estimator have the same expected value and variance. The use of the analytical approach is favourable due to the reduced computational time that results from obviating the Monte Carlo sampling performed by the MCnc for every voxel.

To calculate the noise ceiling on a test data set using the analytical solution it is necessary to estimate from the fMRI time series the variance of the response to the stimuli. Following the standard univariate approach, a parametric estimate of the variance of the responses can be obtained based on an estimation of the noise covariance matrix. Otherwise, a non-parametric estimate of the variance can be obtained by considering the variance of the responses across fMRI runs (and or sessions). In simulations, we showed that such an approach is robust to different types of noise (i.i.d., autocorelated noise and non-stationary noise) as well as to random variations of the responses (caused by factors such as fatigue) that cannot be accounted by the computational model. Note that this estimate of the variance can also be used in the MCnc estimator that originally used a parametric estimate of the variance based on the i.i.d. assumption for the noise [16].

In simulations we showed that the introduced analytical NC using the run to run variability results in similar noise ceiling estimates to the split half noise ceiling (SHnc), which is based on two independent estimates of the test data responses. In real data the SHnc showed slightly higher NC values than the R2Rnc systematically across the 10 subjects. Two reasons can explain this discrepancy: the reduced number of runs used for computing the run to run $\hat{\beta }$ variances (every sound was presented only in six fMRI runs in this experiment, see section MRI data), or violations in the independence between trials which is assumed by the SHnc when accounting for the reduced amount of data in each half. In light of this discrepancy it may be preferable to use the most conservative estimate of the noise ceiling.

Computational models of cognitive processes can consider a high number of parameters. In these cases regularization can be used to avoid overfitting or collinearity of the stimuli in the model parameter space. Our simulations indicate that, when using regularization, the predicted responses using the true computational model (i.e. the model used to simulate the data) cannot reach the noise ceiling.

It is important to stress that, the noise ceiling is itself a random variable with its corresponding variability. The expected value corresponds to the maximum performance conditioned to the noise in the test data. However, for a particular data sample it is possible that the performance of a model being tested to be above the noise ceiling estimate on the same test set. Our results show that different estimators have different variability with the split half estimator exhibiting the larger variance, which is a reason for preferring an analytic (or Monte Carlo) estimate that uses run to run variability as an estimate of the variance of the responses. These considerations also grant a comment on the practice of reporting noise corrected performance values (see e.g. [37]). An evident advantage of this practice is that the noise corrected accuracies allow a direct comparison across experiments (or ROIs) with different levels of noise (e.g. acquired across laboratories or changing MRI acquisition parameters). Nevertheless, considering that the noise ceiling itself is not an observable quantity that is estimated from the data with uncertainty (which is not independent of the mean), this practice is controversial. Different noise ceiling estimators have different variances and rely on different assumptions, and thus at the very least the exact procedure for the estimation of the noise ceiling should be specified. Reporting only noise ceiling corrected performances does not allow assessing (independently) the quality of the data and the observed effect size. Consider the following example: a voxel which reached an accuracy of 0.1 with an estimated NC of 0.2 would result in identical noise corrected accuracy ($\frac{0.1}{0.2}=0.5$) to a voxel which reached 0.4 with NC of 0.8 ($\frac{0.4}{0.8}=0.5$). However the statistical significance and the effect size of an accuracy of 0.1 vs an accuracy of 0.4 differs in orders of magnitude and is computed on the uncorrected accuracies values since the null distribution of the noise normalized accuracies has not been determined yet. For these reasons, it seems more appropriate to report both the observed accuracies and the noise ceiling (see e.g. [38–40]).

The framework proposed in this article is based on a two-level procedure for the estimation of the weights of the encoding models (first responses are estimated from the fMRI time series and then the model is fit to the estimated responses). One conceptual advantage of this two-level approach is that the residual variance can be partitioned in the variance of the experimental noise (level I) and the residual variance after fitting the model (level II). Other approaches directly fit the encoding model convolved with the haemodynamic response to the fMRI time series [3]. The interplay between the haemodynamic response and the computational model makes it difficult to assess the experimental variability of the fMRI time series and as a consequence the noise ceiling. One solution would be to report both accuracies and noise ceiling after the removal of the hemodynamic effect. Alternatively, the SHnc can be used for the calculation of the NC at the level of the whole fMRI time series. Finally, here we evaluated approaches to compute noise ceiling at the single subject level for single voxel encoding models. Some of these approaches (e.g. the SHnc) can be extended to calculate noise ceiling for representational similarity analysis (RSA) always at the single subject level. For group level analysis the approaches we have described here are not directly suitable because they only account for the measurement error and not for the variability between subjects. For RSA, a method has been proposed to compute the noise ceiling at the group level [41] and can be extended to encoding approaches [15].

### Data availability statement

The data and the matlab codes are achieved and described at zenodo.org. The voxels time series for one subject, the fMRI design matrix, the $\hat{\beta }$ images (assuming $\hat{\Omega }=I$) and the Matlab codes for computing the noise ceiling can be downloaded from the same zenodo url at: https://zenodo.org/deposit/1489531, doi: 10.5281/zenodo.1489531. Additional data and codes can be obtained by request to the authors (a.lagecastellanos@maastrichtuniversity.nl).

## Supporting information

S1 Text Relationship between the correlation coefficient and R2. Derivation of the analytical noise ceiling.
(DOCX)

S1 FigNoise ceiling estimates obtained in real fMRI data the inclusion of GLMdenoise noise regressors.Histograms represent the noise ceiling obtained for 50000 voxels (randomly selected) for one subject. Every row in the figure presents the noise ceiling estimated under different parametrizations of the noise covariance matrix (from top to bottom: $\hat{\Omega }=I,\;{\hat{\Omega }}_{AR(1)}$ and ${\hat{\Omega }}_{NST}$). The R2Rnc and the SHnc are presented in the two right most columns and, in each row, are computed based on the $\hat{\beta }$ obtained under different parametrizations of the noise covariance matrix.(TIF)

S2 Fig Histograms of the noise ceiling obtained without the inclusion of the GLMdenoise regressors for ten subjects. The split half method (blue) and the analytical solution (R2Rnc) were computed in 50000 (randomly selected) voxels for 10 subjects.
(TIF)
