# Supplementary material for: Methods for computing the maximum performance of computational models of fMRI responses
Source: PLoS Comput Biol. 2019 Mar 8;15(3):e1006397. doi: 10.1371/journal.pcbi.1006397 (PMC6426260; doi:10.1371/journal.pcbi.1006397)
Supplement: S1 Text — Derivation of the analytical noise ceiling. (DOCX) [file pcbi.1006397.s001.docx]

**Supplementary Information: Relationship between the correlation coefficient and R2**

The explained variance, under the assumption that the observations were normalized to have zero mean $\left( \bar{\hat{\beta}}=\frac{1}{n}\sum_{i} \hat{\beta}_{i}=0 \right)$ is:

|  | $R^{2}=1-\frac{\sum_{i}^{n} \left( \hat{\beta}_{i}-\beta_{i}^{*} \right)^{2}}{\sum_{i}^{n} \hat{\beta}_{i}^{2}}=-\frac{\sum_{i}^{n} {\beta_{i}^{*}}^{2}}{\sum_{i}^{n} \hat{\beta}_{i}^{2}}+2\frac{\sum_{i}^{n} \hat{\beta}_{i}\beta_{i}^{*}}{\sum_{i}^{n} \hat{\beta}_{i}^{2}}$ | (1) |
| --- | --- | --- |

The correlation coefficient under $\bar{\hat{\beta}}=0$ is:

|  | $\rho=\frac{\sum_{i}^{n} \hat{\beta}_{i}\left( \beta_{i}^{*}-\bar{\beta}^{*} \right)}{\sqrt{\left( \sum_{i}^{n} \hat{\beta}_{i}^{2} \right)\sum_{i}^{n} \left( \beta_{i}^{*}-\bar{\beta}^{*} \right)^{2}}}$ | (2) |
| --- | --- | --- |

Writing $R^{2}$as a function of ρ produces

|  | $R^{2}=-\frac{\sum_{i}^{n} {\beta_{i}^{*}}^{2}}{\sum_{i}^{n} \hat{\beta}_{i}^{2}}+2\rho\sqrt{\frac{\sum_{i}^{n} \left( \beta_{i}^{*}-\bar{\beta}^{*} \right)^{2}}{\sum_{i}^{n} \hat{\beta}_{i}^{2}}}$ | (3) |
| --- | --- | --- |

Based on the following the property of the unbiased estimator of the variance:

$\sum_{i} \beta_{i}^{2}=\left( n-1 \right)\hat{\sigma}_{\beta}^{2}+n\bar{\beta}^{2}$ the relation between $R^{2}$ and $\rho$ becomes:

|  | $R^{2}=2\rho\frac{\hat{\sigma}_{\beta^{*}}}{\hat{\sigma}_{\hat{\beta}}}-\frac{\hat{\sigma}_{\beta^{*}}^{2}}{\hat{\sigma}_{\hat{\beta}}^{2}}-\frac{{n \bar{\beta}^{*}}^{2}}{{(n-1)\hat{\sigma}}_{\hat{\beta}}^{2}}$ | (4) |
| --- | --- | --- |

The assumption of using responses that are centered (normalized to have zero mean $\left( \frac{1}{n}\sum_{i} \hat{\beta_{i}}=0 \right)$ ) reflects the interest of encoding models in describing the variations in the brain response around their mean value as a function of the differences between the features of the presented stimulus. In the more general scenario where $\hat{\boldsymbol{\beta}}$ is non-centered the term $\frac{{n \bar{\beta}^{*}}^{2}}{{(n-1)\hat{\sigma}}_{\hat{\beta}}^{2}}$ becomes into $\frac{n\left( \bar{\beta}^{*} - \bar{\hat{\beta}} \right)^{2}}{{(n-1)\hat{\sigma}}_{\hat{\beta}}^{2}}$ in the previous formula.

**Supplementary Information: Derivation of the analytical noise ceiling**

The definition of noise ceiling for a vector of responses $\hat{\boldsymbol{\beta}}$and vector of responses of true brain responses $\boldsymbol{\beta}$ (not directly observed from the data) is (Eq14):

|  | $\rho_{\mathrm{NC}}=E\left( \rho\right)_{\boldsymbol{\beta}^{\boldsymbol{*}}\boldsymbol{=\beta}}$  $\rho_{\mathrm{NC}}=\frac{\frac{1}{(n-1)}\sum_{i}^{n} \left( \hat{\beta}_{i}-\bar{\hat{\beta}} \right)\left( \beta_{i}-\bar{\beta} \right)}{\sqrt{{\sigma_{\hat{\boldsymbol{\beta}}}^{2}\sigma}_{\boldsymbol{\beta}}^{2}}}$ | (5) |
| --- | --- | --- |

This definition refers to the covariation across the components $i$ of the “noise free response” $\boldsymbol{\beta}$ and the estimated response $\hat{\boldsymbol{\beta}}$ (noise contaminated). Note that each component of the $\hat{\beta}_{i}$can be written as: $\hat{\beta}_{i} =\beta_{i}+\varepsilon_{i}$, where $\varepsilon_{i}$ the variability of each component of $\hat{\beta}$ around the expected value due to experimental noise. The variance of $\varepsilon_{i}$ derived is $\hat{\sigma}_{\varepsilon_{i}}^{2}=\hat{V}_{ii}$, (derived from one of the methods presented in this article. e.g GLS) where the index *ii* refers to *i* the diagonal entries of the corresponding covariance matrix. Substituting $\hat{\beta}_{i} =\beta_{i}+\varepsilon_{i}$ into the NC definition produce:

|  | $\rho_{\mathrm{NC}}=\frac{\frac{1}{(n-1)}\sum_{i}^{n} \left( \beta_{i}-\bar{\beta}+\varepsilon_{i}-\bar{\boldsymbol{\varepsilon}} \right)\left( \beta_{i}-\bar{\beta} \right)}{\sqrt{\sigma_{\hat{\beta}}^{2}\sigma_{\beta}^{2}}}$ | (6) |
| --- | --- | --- |

Noticing that, (for unbiased estimators of $\boldsymbol{\beta}$) the expected value of the estimation error each $\varepsilon_{i}$ is 0, and that $\varepsilon_{i}$and $\beta_{i}$ are independent. The expected value of the noise ceiling becomes:

|  | $\rho_{\mathrm{NC}}=\frac{\frac{1}{(n-1)}\sum_{i=1}^{n} \left( \beta_{i}-\bar{\beta} \right)^{2}}{\sqrt{\sigma_{\hat{\beta}}^{2}\sigma_{\beta}^{2}}}=\frac{\sigma_{\beta}}{\sigma_{\hat{\beta}}}$ | (7) |
| --- | --- | --- |

The estimator of $\rho_{NC}$ is obtained by substituting $\sigma_{\beta}$ and $\sigma_{\hat{\beta}}$ by its corresponding estimators. The estimator of $\sigma_{\hat{\beta}}$ is $\hat{\sigma}_{\hat{\left( \beta\right)}}=\frac{1}{n-1}\sum_{i} \left( \hat{\beta}_{i}-\bar{\hat{\beta}} \right)^{2}$ . Consider that if $\beta_{i}=\beta_{i}+\varepsilon_{i}$ and $\beta_{i}$ and $\varepsilon_{i}$ are independent, then: $\sigma_{\hat{\beta}}^{2}=\sigma_{\beta}^{2}+\sigma_{\varepsilon}^{2}$. The estimator of $\sigma_{\beta}^{2}$ is:

|  | $\hat{\sigma}_{\beta}^{2}=\hat{\sigma}_{\hat{\beta}}^{2}-\hat{\sigma}_{\varepsilon}^{2}$ | (8) |
| --- | --- | --- |

With: $\hat{\sigma}_{\varepsilon}^{2}=\frac{1}{n}\sum_{i=1}^{n} \left( \hat{V}_{ii} \right)$. Substituting these estimators into the noise ceiling formula we obtain that the estimator of the noise ceiling is:

|  | $\hat{\rho}_{NC}=\frac{\sqrt{\hat{\sigma}_{\hat{\beta}}^{2}-\frac{1}{n}\sum_{i=1}^{n} \hat{V}_{ii}}}{\hat{\sigma}_{\hat{\beta}}}$ | (9) |
| --- | --- | --- |
