# Supplementary figures and images for: Methods for computing the maximum performance of computational models of fMRI responses
Source: PLoS Comput Biol. 2019 Mar 8;15(3):e1006397. doi: 10.1371/journal.pcbi.1006397 (PMC6426260; doi:10.1371/journal.pcbi.1006397)

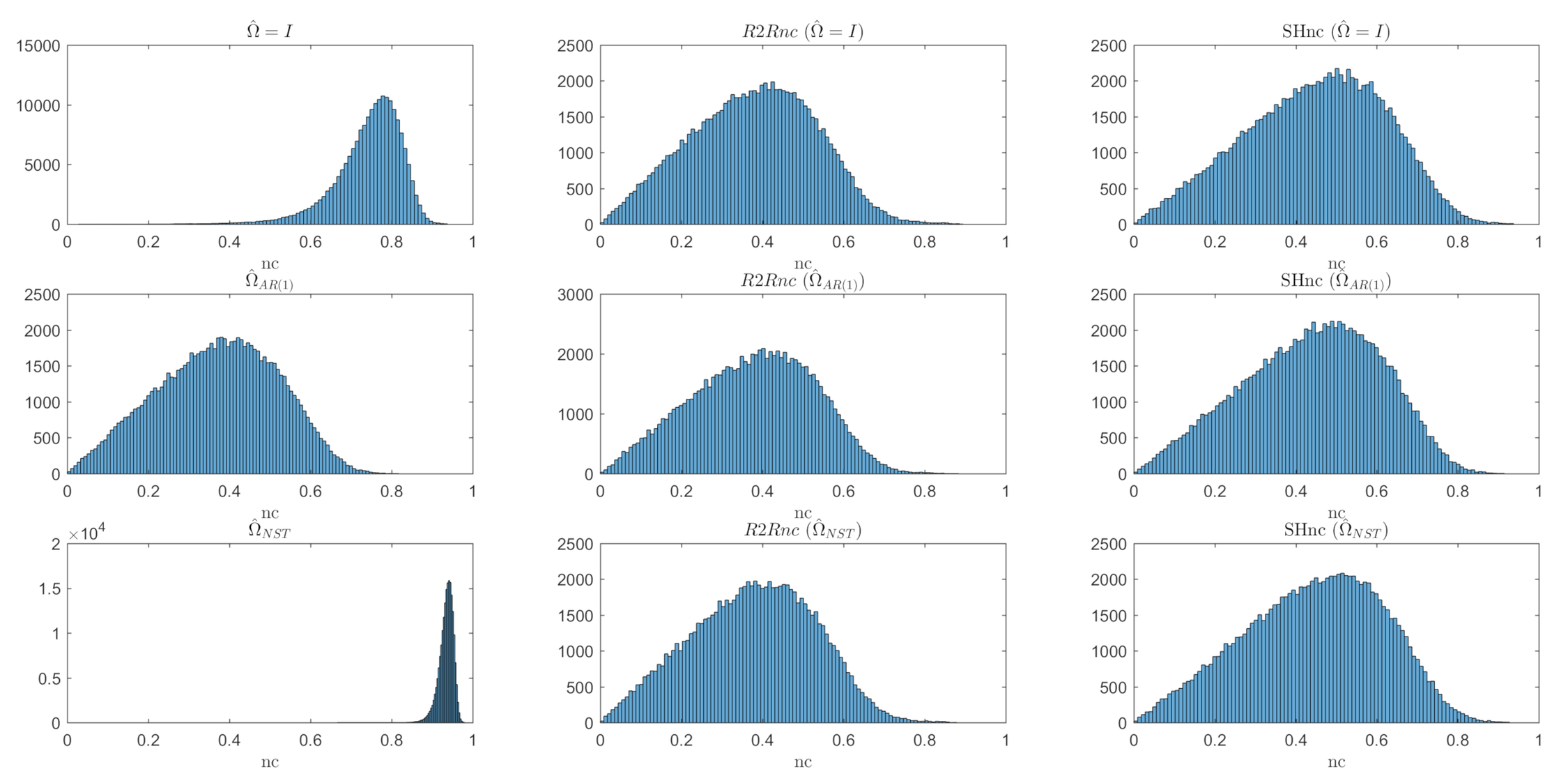

Supplement: S1 Fig — Histograms represent the noise ceiling obtained for 50000 voxels (randomly selected) for one subject. Every row in the figure presents the noise ceiling estimated under different parametrizations of the noise covariance matrix (from top to bottom: Ω^=I,Ω^AR(1) and Ω^NST). The R2Rnc and the SHnc are presented in the two right most columns and, in each row, are computed based on the β^ obtained under different parametrizations of the noise covariance matrix. (TIF) [file pcbi.1006397.s002.tif]

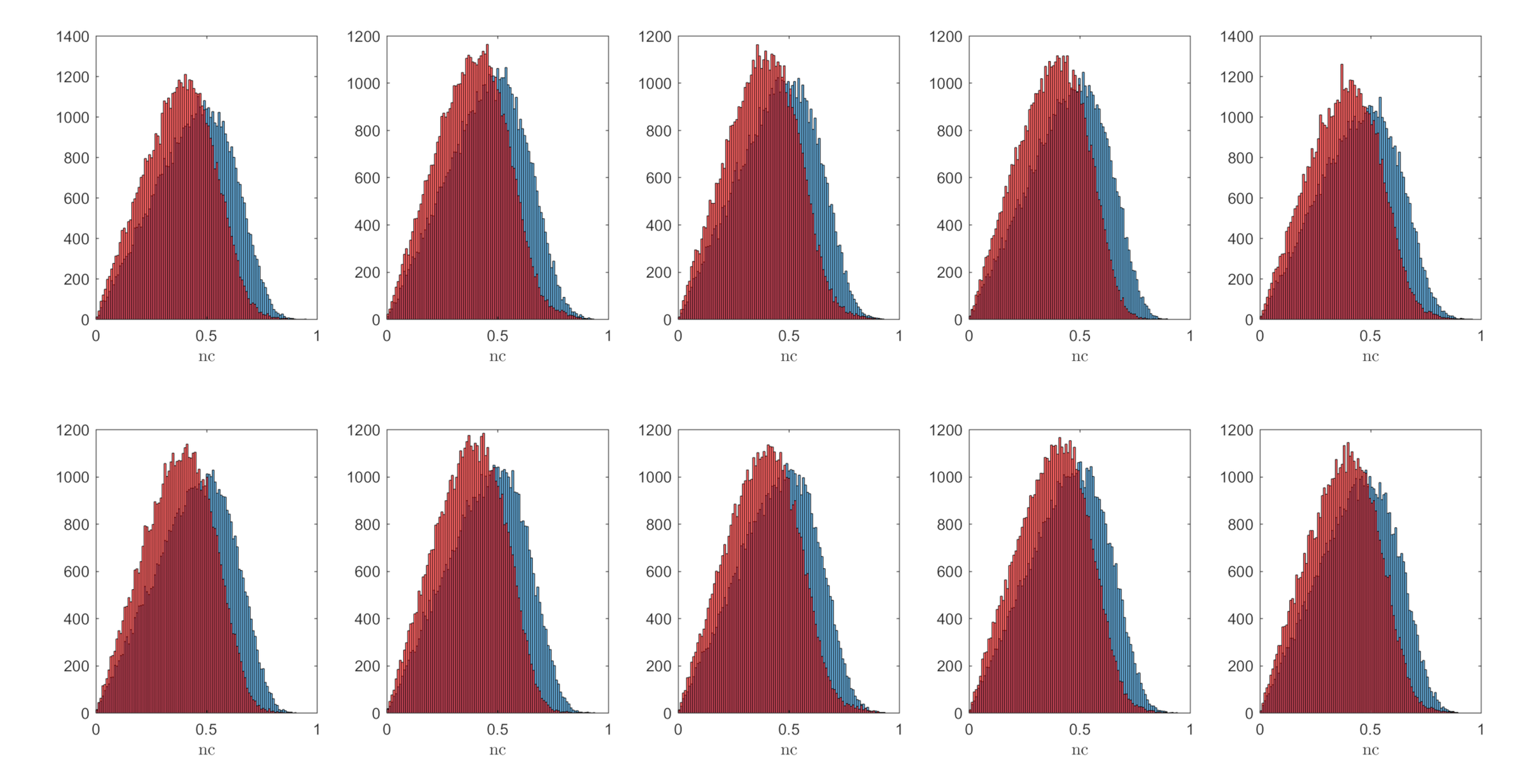

Supplement: S2 Fig — The split half method (blue) and the analytical solution (R2Rnc) were computed in 50000 (randomly selected) voxels for 10 subjects. (TIF) [file pcbi.1006397.s003.tif]
